# Pharmacological CLK inhibition disrupts SR protein function and RNA splicing blocking cell growth and migration in TNBC

**DOI:** 10.1186/s13058-025-02091-w

**Published:** 2025-07-29

**Authors:** Nasi Liu, Jurjun J. S. van der Velde, Sherien Ramdjielal, Esmee Koedoot, Nila K. van Overbeek, Daisy Batenburg, Alfred C. O. Vertegaal, Bob van de Water, Sylvia E. Le Dévédec

**Affiliations:** 1 Division of Cell Systems and Drug Safety, Leiden Academic Centre for Drug Research, Leiden University, Einsteinweg 55, Leiden, 2333 CC The Netherlands; 2 Department of Cell and Chemical Biology, Leiden University Medical Center, Albinusdreef 2, Leiden, 2333 ZA The Netherlands

**Keywords:** Triple-negative breast cancer, Alternative splicing, Cdc2-like kinase, Serine/arginine-rich proteins

## Abstract

**Background:**

Dysregulation of alternative splicing plays a pivotal role in tumorigenesis and metastasis in triple-negative breast cancer (TNBC). Serine/arginine-rich (SR) proteins, essential components of the spliceosome, undergo phosphorylation by Cdc2-like kinase (CLK). Here we explored the impact of pharmacological inhibition of CLK using a novel inhibitor, T-025, on the spliceosome complex and transcriptional responses in relation to cell proliferation and migration in TNBC.

**Methods:**

We evaluated the anti-proliferative and anti-migratory efficacy of T-025 in a spectrum of TNBC cell lines. Fluorescent reporter cell lines and flowcytometry were used to determine the effect of T-025 on cell cycle. Deep RNA sequencing was performed to unravel the differentially expressed genes (DEGs) and alternatively spliced genes (ASGs) upon T-025 treatment. Pulldown/MS was used to uncover the impact of T-025 on SRSF7 interactome. Live-cell imaging and photobleaching experiments were conducted to determine the subnuclear localization of SRSF7-GFP and its dynamic mobility.

**Results:**

T-025 exhibited a potent anti-proliferative effect in a spectrum of TNBC cell lines, particularly in highly proliferative cell lines. Treatment with T-025 induced cell cycle arrest in the G1-S phase, resulting in an increased proportion of aneuploidy cells and cells with 4 N DNA. T-025 significantly inhibited cell migration in highly migratory TNBC cell lines. Deep RNA sequencing uncovered numerous DEGs and ASGs upon T-025 treatment, which were significantly enriched in pathways related to cell division, RNA splicing and cell migration. Pulldown/MS showed that SRSF7 interacted more with nuclear-speckle-residing proteins, while less with RNA helicases and polymerases upon T-025 treatment. Enhanced interactions between SRSF7 and other phosphorylated SR proteins localized at nuclear speckles were also observed. Live-cell imaging indicated that T-025 treatment induced the accumulation of SRSF7-GFP at nuclear speckles and nuclear speckles’ enlargement, restricting its protein dynamic mobility.

**Conclusions:**

CLK inhibition using T-025 leads to the accumulation of splicing factors at nuclear speckles and stalls their release to splicing sites, resulting in the RNA splicing reprogramming of a large number of genes involved in cell division, migration and RNA splicing. Our findings provide evidence that T-025 could be a promising therapeutic drug for TNBC patients.

**Supplementary Information:**

The online version contains supplementary material available at 10.1186/s13058-025-02091-w.

## Introduction

Triple-negative breast cancer (TNBC) is the most aggressive breast cancer subtype and associated with a higher risk of recurrence and distant metastasis. Although TNBC accounts for only 15%-20% of all breast cancer (BC) cases, it disproportionally contributes to BC mortality [1]. Due to a lack of estrogen receptor (ER), progesterone receptor (PR) and human epidermal growth factor receptor 2 (HER2), hormone therapy is ineffective in TNBC, leaving patients with limited treatment options and no targeted therapies available yet in the clinic [2]. Patients with TNBC still mostly rely on traditional chemotherapy, but resistance often develops after the initial response. Despite numerous attempted therapies and clinical trials, no significant survival benefits have been observed [3–5]. Therefore, novel and effective targeted therapeutic strategies for TNBC are urgently needed.

RNA splicing is a complex process that removes the non-coding RNA (introns) from primary transcripts, essential for mRNA maturation [6]. In addition to constitutive splicing, many genes undergo alternative splicing (AS), a critical posttranscriptional regulatory process that selectively includes exons and introns [7]. RNA-sequencing data reveals that nearly 94% of the genes can be alternatively spliced, resulting in transcriptome and proteome diversity in eukaryotes [8]. Dysregulation of AS plays a key role in tumorigenesis and cellular plasticity [9]. Hence, RNA-based therapies offer the potential to modulate splicing changes in cancer cells, enhancing their sensitivity to traditional chemotherapy [10–13].

Serine and arginine-rich (SR) proteins are regulatory nuclear RNA-binding proteins (RBPs) that play a crucial role in RNA splicing [14]. These proteins contain at least one N-terminal RNA recognition motif (RRM) and a C-terminal RS domain spanning at least 50 amino acids, with serine and arginine content exceeding 40%. The RRM domain recognizes the RNA binding site in pre-mRNA, while the RS domain facilitates spliceosome assembly and the shuttling of specific SR proteins between the nucleus and cytoplasm. This shuttling process is highly dependent on phosphorylation status [15–17]. Currently, three predominant protein kinase families regulate the phosphorylation of SR proteins: (i) SR protein-specific kinases (SRPKs), (ii) pre-mRNA processing factors (PRPFs) and (iii) Cdc2-like kinases (CLKs) [17, 18]. In our previous study, we have unravelled the critical role of SRPK1 and PRPF4B in breast cancer progression and metastasis [19, 20]. Emerging studies have also demonstrated the importance of CLKs in many cancer types and they could be novel anti-cancer therapeutic target [21–26]. The CLK family of kinases comprises four homologues: CLK1, CLK2, CLK3, and CLK4. They share a common structural framework, featuring a C-terminal kinase domain, which exhibits high conservation across all family members, and an N-terminal domain which characterized by the abundance of arginine/serine (RS) dipeptides whereas varies among all CLKs. The RS-rich N-terminal domain is considered as a bridge mediating the interaction between their C-terminal kinase domain and the RS domains of SR protein, which is well-characterised substrate of CLKs that facilitates spliceosome assembly upon phosphorylation by CLKs [27].

Given the essential role of CLKs in cancer, small molecule inhibitors have been developed and provide avenues for cancer treatment [23, 28, 29]. SM08502 is the first small molecule inhibitor of CLKs which entered Phase 1 first-in-human study, but the study was terminated due to business reasons by sponsor [30]. T-025, a potent and orally available CLK inhibitor identified recently, exhibits strong affinity to CLK family, showing more than 300-fold enhanced selectivity compared with other kinases, even though it also potentially inhibits DYRK1. Additionally, in contrast to other CLK inhibitors such as TG003, DB18, CLK-IN-T3 and MU1210 which display relatively weak inhibitory activity against CLK3 or CLK4, T-025 shows low dissociation constant (*K*_d_) value for all CLK family members [31–34]. Furthermore, It was suggested that T-025 reduced SR proteins phosphorylation and inhibited cell proliferation in various solid cancer cell lines, especially MYC-driven cancers [35]. However, the impact of T-025 on SR protein activity and alternative splicing in TNBC, particularly in highly migratory TNBC cell lines, remains under-investigated.

In the present study, we explored the impact of pharmacological inhibition of CLK using a novel inhibitor, T-025, on cell proliferation across a spectrum of TNBC cell lines and on cell migration in two highly migratory TNBC cell lines. Serine/arginine-rich splicing factor 7 (SRSF7) is a key spliceosome component, which was recently identified as a critical player in controlling alternative splicing during TNBC progression in our previous study (unpublished). We found that CLK inhibition by T-025 led to an accumulation of the SRSF7 within nuclear speckles, thereby limiting its dynamic mobility. This restriction in turn led to aberrant RNA splicing of a large number of genes, especially genes related to RNA splicing, cell division and migration. The ultimate consequence of this aberrant splicing was the induction of mitotic defects and a stop in the migratory capacity of TNBC cells.

## Materials and methods

### Cell culture

MDA-MB-231, Hs578T, SKBR7, HCC1937, SUM159PT, HCC38, HCC1806, MDA-MB-468, BT549, HCC70, SUM149PT, MDA-MB-436, SUM185PE, SUM1315M02, MDA-MB-157 and MDA-MB-453 were kindly provided by Prof. J. Martens from the Erasmus Medical Center. All the cell lines were grown in RPMI-1640 medium (Gibco, Life Technologies, USA) supplemented with 10% fetal bovine serum (FBS), 100 U/ml penicillin (Invitrogen) and 100 mg/ml streptomycin (Invitrogen). Cells were cultured at 37 °C in a humidified 5% CO_2_ incubator.

### Antibodies and reagents

Rabbit anti-CLK2 (HPA055366), Rabbit anti-SRSF7 (HPA056926) were purchased from Atlas antibodies. Rabbit anti-PRPF4B (#8577s), rabbit anti-phospho-Rpb1 (Ser2) (#13499S), mouse anti-Rpb1 (#2629S) and rabbit anti-GFP (#2956) were obtained from Cell Signaling Technology. Mouse anti-tubulin (T-9026) and mouse anti-phospho epitope SR proteins (MABE50), clone 1H4, were purchased from Sigma-Aldrich. Mouse anti-sc35 antibody (S4045, Sigma-Aldrich) was a kind gift from Dr. Vered Raz at Leiden University Medical Centre. Anti-mouse horseradish peroxidase (HRP), anti-rabbit HRP and anti-mouse Alexa-647 conjugated secondary antibodies were purchased from Jackson Immunoresearch Laboratories. T-025 (HY-112296) was purchased from MedChemExpress.

### Transient siRNA mediated gene knockdown

The siRNA SMARTPool (containing four different siRNAs for one gene) targeting CLK1, CLK2, CLK3, CLK4 were purchased from Dharmacon. Reverse transfection was performed using 25 nM siRNA and INTERFERin (Polyplus, Illkirch) according to the manufacture’s guidelines. After 18 h, medium was replaced by complete medium. Cell were used for random cell migration (RCM) assay 72 h after transfection. Transfection reagent only (Mock) or a mixture of 720 siRNAs targeting different kinase genes (non-specific kinase pool, siKinasePool), thereby not significantly affecting any gene expression, were used as negative control.

### Generation of fluorescent reporter cell lines

Human SRSF7, SNRPD2 and SNRPD3 BAC clones were selected and GFP-tagged as previously described [36, 37]. A bacterial artificial chromosome (BAC) containing the gene of SRSF7, SNRPD2 or SNRPD3 and endogenous cis-regulatory elements was tagged with green fluorescent protein (GFP) at the C- terminus. SNRPD2 and SNRPD3 BAC-GFP clones were stably transfected into Hs578T cells. SRSF7 BAC-GFP clone was transected into both Hs578T and MDA-MB-231 cells. Cells were selected using 50 mg/ml G-418 and sorted twice for GFP expression. MDA-MB-231 and Hs578T cell lines with stable integration of the Fluorescent Ubiquitination-Based Cell Cycle Indicator (FUCCI) reporter construct were established using FUCCI plasmid pLL3.7 m-Clover-Geminin(1-110)-IRES-mKO2-Cdt(30–120) for lentiviral transduction. FUCCI plasmid was a kind gift from Michael Lin (Addgene plasmid #83841). Cells expressing the FUCCI construct were subsequently selected for uniformity using fluorescence-activated cell sorting (FACS).

### Sulforhodamine B cell viability assay

Triple-negative breast cancer cell lines were seeded in 96-well plates (655180, Greiner Bio-One) and treated with 0.01, 0.1, 1, 10, 100, 1 000 and 10 000 nM T-025 the following day. After 72 h, cells were fixed by adding 30 ml 50% trichloroacetic acid (TCA) directly into the wells containing 100 µl of medium, and incubated for 1 h at 4 °C. Plates were washed at least five times with distilled water and air-dried overnight. Cells were then stained with 60 µl 0.4% sulforhodamine B (SRB) in 1% acetic acid for 30 min at room temperature, followed by five washes with 150 µl 1% acetic acid to remove the unbound SRB and air-dried overnight. Bound SRB was solubilized by adding 150 µl 10 mM unbuffered Tris to the wells and shaking for 1 h at room temperature. The SRB absorbance was measured on Bio-Rad microplate reader at 540 nm. The IC_50_ of T-025 were calculated using GraphPad Prism version 9.0.0. Cell proliferation rates were determined by calculating the ratio of absorbance at day 3 to day 0 post-treatment with 0.1% DMSO in each cell lines. The correlation curve and Pearson correlation coefficient were also generated using GraphPad Prism version 9.0.0.

### Random cell migration assay

Cells were seeded in µClear black-bottom 96-well imaging plates (655090, Greiner Bio-One) pre-coated with 20 mg/ml rat tail collagen I (354236, Corning) in PBS. Seeding densities were 8000 cells/well for MDA-MB-231 and 6000 cells/well for Hs578T cell line. The following day cells were treated with 100, 200, 500 and 1000 nM T-025. At 24 h post-treatment, cells were stained with Hoechst-33342 for 1 h at 37 °C. Imaging was performed using a 20x objective (0.75 NA, 1.00 WD) on a Nikon Eclipse Ti microscope, equipped with a humidified 37 °C incubation chamber and 5% CO_2_ flow. Images were captured with a DS-Qi1MC CCD camera at 15-minute intervals for 15 h, with 4 positions per well being recorded using NIS-Elements software (Nikon, Amsterdam, the Netherlands). Images were converted to TIFF format and analyzed using Cell Profiler version 2.1.1 and custom R scripts.

### IncuCyte live-cell imaging

MDA-MB-231 and Hs578T cells were seeded in 96-well plates (655180, Greiner Bio-One), with 6000 cells/well for MDA-MB-231 and 5000 cells/well for Hs578T cell line. The following day cells were treated with 100, 200, 500 and 1000 nM T-025. Imaging was conducted immediately using IncuCyte S3 live-cell analysis system (Sartorius). Images were captured at 12-hour intervals for 72 h, with 4 positions per well being recorded. The images were analyzed using IncuCyte 2020B software (Sartorius) to determine the cell confluence at 24, 48 and 72 h post-treatment.

### Cell cycle analysis

MDA-MB-231 and Hs578T FUCCI reporter cells were seeded in a µClear black-bottom 96-well imaging plate, with 6000 cells/well for MDA-MB-231 and 5000 cells/well for Hs578T cell line. The following day, the cells were stained with Hoechst-33342 and subsequently treated with 50, 100, 200, 500 and 1 000 nM T-025. Live cell imaging was conducted immediately using a 20x objective (0.75 NA, 1.00 WD) on a Nikon Eclipse Ti microscope, equipped with a humidified 37 °C incubation chamber and 5% CO_2_ flow. Images were captured with a DS-Qi1MC CCD camera at 6-hour intervals for 48 h, with 4 positions per well being recorded using NIS-Elements software. The images were analyzed using Cell Profiler version 4.2.5 to determine the percentage of cells in different phases of the cell cycle relative to the total cell count. We defined Cdt1-positive cells (detected in the green channel) as being in the G1 phase, Geminin-positive cells (detected in the red channel) as being in the S-G2-M phases, cells positive for both Cdt1 and Geminin- as being in the S phase and cells negative for both but with a Hoechst signal as undergoing the transition from M to G1 phase.

### Propidium iodide (PI)/flow cytometry analysis

Cells were seeded in 10-cm Petri dishes, with a seeding density of 2 million/dish for MDA-MB-231 and 1.5 million/dish for Hs578T. The following day, the cells were treated with 200, 500 and 1 000 nM T-025. After 48 h, cells were collected by trypsinization and centrifugation at 1000 rpm for 5 min. The cell pellet was washed once with PBS and centrifuged to remove PBS, then fixed by adding 1 ml 70% ice-cold ethanol dropwise under gentle vortexing and stored at 4 °C for 2 h. Cells were then washed twice with PBS and resuspended in 200 µL PBS with 5 µL 10 mg/ml RNase A, then incubated for 1 h in a water bath at 37 °C. 10 µL 1 mg/ml PI was directly added into the cell suspension to achieve a final PI concentration of 50 µg/ml, and the suspension was kept in the dark at room temperature for 1 h. Cells were filtered through 45 μm filter to achieve single cells before detection on the CytoFLEX flow cytometer (Beckman Coulter). DNA content and aneuploidy cell population were analyzed using Modfit LT version 6.0.

### Live cell imaging of SRSF7 subnuclear localization in SRSF7-GFP reporter cell lines

BAC-SRSF7-GFP reporter cells were seeded in glass-bottom Sensoplate 96-well imaging plate (655892, Greiner Bio-One) coated with 20 mg/ml rat tail collagen I (354236, Corning) in PBS. The seeding density was 15,000/well for the Hs578T cell line. The following day, cells were stained with Hoechst-33342 and treated with 1 µM T-025. Live cell imaging was performed using a 20x objective (0.75 NA, 1.00 WD) on a Nikon Eclipse Ti microscope, equipped with a humidified 37 °C incubation chamber and 5% CO_2_ flow. Images were captured with a DS-Qi1MC CCD camera at 30-minute intervals for 25 h, with 4 positions per well being recorded using NIS-Elements software. Images were analyzed in Cell Profiler version 4.2.5 (Suppl. Figure 1 A), where the areas of SRSF7-GFP in speckles and in the nucleoplasm were identified, respectively. The count and occupied area of SRSF7-GFP accumulated speckles were measured. Meanwhile, the integrated intensity of SRSF7-GFP which is the sum of the pixel intensities within the segmented areas of interest: speckles and nucleoplasm were quantified as well. To eliminate the interference of GFP negative and overexpressed cells, all the measurements were made after correcting for nuclei count with GFP expression and filtering out the cells with overexposed GFP whose GFP integrated intensity were above a certain threshold. All the images and the analysis pipeline in Cell Profiler were submitted to BioImage Archive with the accession number S-BIAD1498 [38].

### Photobleaching experiments

BAC-SRSF7-GFP Hs578T and MDA-MB-231 cells were seeded in glass-bottom Sensoplate 96-well imaging plate (655892, Greiner Bio-One) coated with 20 mg/ml rat tail collagen I in PBS, and treated with 1 µM T-025 the following day. After 24 h, cells were stained with Hoechst-33342 and imaged at 37 °C using a PlanApo VC 60×/1.40 NA Oil objective on Nikon A1 confocal microscope equipped with a humidified 5% CO_2_ incubator. The imaging and photobleaching were conducted using NIS-Elements software. Briefly, half of the nucleus region of cells with medium GFP intensity was selected as “Stimulation ROI” and another half of nucleus region was selected as “Background ROI”. Then the “Stimulation ROI” was bleached for 6 s under the highest laser power in A1plus Stimulation panel after a pre-scan to sufficiently remove the soluble SRSF7-GFP. This caused a slight reduction in fluorescence intensity in the remaining unbleached region (Background ROI) as well. After photobleaching, images were acquired every 5 s for 2.5 min to allow complete equilibration of soluble SRSF7-GFP across the nucleus. Fluorescence intensity in the bleached (Stimulation ROI) and unbleached region (Background ROI) was measured throughout the entire procedure and exported on NIS-Elements software. Ultimately the normalized fluorescence intensity was analyzed by considering the fluorescence intensity of the pre-bleaching frame as 100% and the post-bleaching frame as 0% in the bleached area. All the photobleaching images and fluorescence intensity measurements generated in NIS-Elements software were submitted to BioImage Archive with the accession number S-BIAD1501 [38].

### Immunofluorescence

The BAC-SRSF7-GFP Hs578T and MDA-MB-231 cells used for live cell imaging were washed once with PBS and fixed with 1% paraformaldehyde (PFA) with 0.1% Triton X-100 in PBS for 20 min at room temperature. This was followed by three washes with PBS. Cells were then blocked with 0.1% bovine serum albumin (BSA) in PBS for 30 min at room temperature. After removal of the BSA-PBS solution, mouse anti-sc35 antibody was added into the wells and incubated overnight at 4 °C. The following day, cells were washed three times with 0.1% BSA-PBS, with each wash lasting 10 min. Cells were then stained with goat anti-mouse secondary antibody conjugated with Alexa 647 for 1 h in the dark at room temperature. After staining, cells were washed once with 0.1% BSA-PBS and twice with PBS, with each wash lasting 10 min. Finally, cells were imaged using a 40x WI objective (1.15 NA, 0.61–0.59WD) on a Nikon Eclipse Ti microscope.

### Immunoprecipitation

MDA-MB-231-GFP and BAC-SRSF7-GFP MDA-MB-231 reporter cells were seeded in eight 10-cm Petri dishes, with a seeding density of 3 million/dish. MDA-MB-231-GFP cells were used as a negative control in the following GFP pull-down procedures. After two days, BAC-SRSF7-GFP MDA-MB-231 reporter cells were treated with 1 µM T-025 or 0.1% DMSO for 24 h. Cells were washed twice with cold PBS, and then scraped off the dishes. This was followed by centrifugation at 1500 rpm for 5 min at 4 °C and removal of the supernatant. Cells were collected and lysed in 300 µl EBC buffer (50 mM Tris pH 7.3, 150 mM NaCl, 0.5% NP-40 and 1 mM MgCl2) containing protease inhibitor mixture (#11697498001, Roche), as well as 5 mM sodium fluoride and 1 mM sodium orthovanadate for phosphatases inhibition. The cell lysate was sonicated six times for 5 s with a 5-second cooling period in between. 500 U benzonase (E1014, Sigma) was added to the cell lysate after adding 700 µl EBC buffer, followed by 1-hour incubation at 4 °C under rotation. NaCl was added to the lysates, to a final concentration of 300 mM. Lysates were centrifuged for 10 min at 13,000 rpm, followed by BCA protein concentration measurement. An equal amount of protein was added to 20 µl GFP-Trap agarose beads (Chromotek) and incubated for 2 h at 4 °C under rotation. For Western blotting, beads were washed six times with 1 ml EBC buffer (with 300 mM NaCl) under rotation for 5 min at 4 °C. 60 µl 2x Laemmli buffer (S3401-10VL, Merck) was added to the beads, and the samples were boiled for 7 min at 95 °C before loading onto the gel.

### Mass spectrometry sample preparation

For mass spectrometry, beads were washed four times with 1 ml EBC buffer (with 300 mM NaCl) and three times with 1 ml 50 mM ammonium bicarbonate. All washes were conducted under 5-minutes rotation at 4 °C. Beads were incubated with 250 µl 50 mM ammonium bicarbonate containing 1 ug trypsin (V5111, Promega) overnight at 37 °C under shaking. The following day, 25% TFA (40967, Sigma) was added to the beads to a final concentration of 1% to stop digestion. Next, samples were centrifuged for 5 min at 6000 rpm and digests were loaded on a prepared tC18 cartridge (washed twice with acetonitrile and 1% acetic acid). Peptides were desalted by washing twice with 1% acetic acid, then eluted with 300 µl 0.1% acetic acid/60% acetonitrile. Finally, peptides were vacuum dried and dissolved in 0.1% formic acid.

### Mass spectrometry data acquisition

GFP pull-down samples were analyzed by online C18 nanoHPLC MS/MS with a system consisting of an Ultimate3000nano gradient HPLC system (Thermo Fisher Scientific, Bremen, Germany), and an Exploris480 mass spectrometer (Thermo Fisher Scientific, Bremen, Germany). Digested peptides were injected onto a cartridge precolumn (300 μm × 5 mm, C18 PepMap, 5 μm) in 100% solvent A (0.1% formic acid in miliQ), with a flow of 10 µl/min for 3 min (Thermo, Bremen, Germany) and eluted via a homemade analytical nano-HPLC column (30 cm × 75 μm; Reprosil-Pur C18-AQ 1.9 μm, 120 A (Dr. Maisch, Ammerbuch, Germany). The chromatography gradient length was 60 min from 2 to 40% solvent B, followed by a 5 min increase to 95% solvent B, another 5 min of 95% solvent B and back to 2% solvent B for chromatography column re-conditioning. The mass spectrometer was operated in positive polarity data-dependent MS/MS mode with a cycle time between master scans of 3 s. Full scan MS spectra were obtained with a resolution of 60,000, a normalized automatic gain control (AGC) target of 300%, and a scan range of 350–1,600 m/z. Precursors were fragmented by Higher-Collisional Dissociation (HCD) with a normalized collision energy of 28%. Tandem mass spectra (MS/MS) were recorded with a resolution of 30,000 and a normalized AGC target value of 75%. Precursor ions selected for MS/MS analysis were subsequently dynamically excluded from MS/MS analysis for 30 s and only precursors with a charge state of 2–6 triggered MS/MS events.

### Mass spectrometry data analysis

Raw mass spectrometry files were analyzed using MaxQuant (version 2.1.3.0) with a database search against an in silico digested reference proteome for Homo sapiens (UniProt, reviewed, canonical, downloaded 5th of September, 2023). Database searched were performed with default settings apart from the following modifications: Phospho (STY) was added as a variable modification and both Label Free Quantification (LFQ) and the match-between-runs feature were enabled (default settings). The output from MaxQuant was further analyzed in Perseus (v1.5.5.3). LFQ intensity values were log2 transformed. Potential contaminants and reverse peptides were removed. Samples were grouped according to the experimental categories, and proteins identified in 3 out of 4 biological replicates in at least one group were included. Missing values were imputed by normally distributed values with a 1.8 downshift (log2) and a randomized 0.3 width (log2), considering total matrix values. Student’s T-tests were performed with a threshold false discovery rate (FDR) of 0.05. The final matrix after analysis was exported for further data processing. Proteins with a threshold of log2 fold change greater than 1 or less than − 1 and an FDR less than 0.05 were identified as upregulated or downregulated SRSF7 interactors respectively upon T-025 treatment. Subsequently, identified downregulated and upregulated interactors were used for gene ontology (GO) enrichment analysis using the DAVID Gene Functional Classification Tool [39]. The mass spectrometry proteomics data have been deposited to the ProteomeXchange Consortium via the PRIDE partner repository with the dataset identifier PXD058273 [40].

### Western blotting

Proteins were separated by electrophoresis on SDS-PAGE gels, followed by transfer to PVDF membranes (Millipore) for 100 min under 100 V on ice. Subsequently, proteins on the membrane were blocked with 5% BSA in TBS buffer containing 0.05% Tween-20 for 1 h at room temperature, then incubated with the corresponding primary antibody overnight at 4 °C. Membranes were washed three times for 10 min on a shaker with TBS buffer containing 0.05% Tween-20, then incubated with secondary antibody for 1 h at room temperature. Following this, membranes were exposed to ECL Prime western blotting detection reagent (RPN2236, Cytiva) and imaged using an Amersham Imager 600 (GE Heathcare). The quantifications of the bands were performed using Fiji ImageJ version v1.51j.

### RNA sequencing library preparation and data analysis

A total of 30 million MDA-MB-231 cells were seeded in a 6-well plate. The following day, cells were treated with 1 µM T-025 or 0.1% DMSO for 24 h. Total RNA was isolated using the RNeasy plus mini kit (Qiagen). Samples from 3 biological replicates were used. RNA quality and quantity were measured on an Agilent-4200 Bioanalyzer. Stranded PolyA-selected libraries were prepared using the DNBseq platform, and 100 M 150-bp paired-end reads were sequenced on a DNBSEQ-G400 sequencer by BGI Europe. Reads were aligned against the human GRCH38 reference genome (gencode version 45) using the STAR aligner (version 2.7.11b). Gene expression quantification was performed concurrently during mapping with the STAR --quantMode option set to GeneCounts. Count data were normalized, and log2 fold change and adjusted p-value were calculated using the DESeq2 package (version 1.44.0). Differentially expressed genes (DEGs) were selected with a threshold of log2 fold change greater than 1 or less than − 1 and an adjusted p-value less than 0.01 and used for GO enrichment analysis using the DAVID Gene Functional Classification Tool [39]. Alternative splicing events were analyzed using rMATS package (version 4.2.0). The significantly changed alternative splicing events (ASEs) were determined by average read counts in two samples (greater than 10), the absolute value of the difference for percentage spliced-in value (|ΔPSI|) (greater than 0.1) and statistical significance of the change (FDR < 0.01). For GO enrichment analysis, we selected the ASEs with|ΔPSI| >0.4 and FDR < 0.01. Bioinformatic analysis was performed using the OmicStudio tools at https://www.omicstudio.cn/tool. The RNA sequencing data have been deposited to ArrayExpress with the accession number E-MTAB-14,664 [38].

### Data analysis and statistics

All experiments were performed in biological triplicates unless indicated. Data were analyzed in GraphPad Prism 9.0, with Student’s T-test (two-tailed, equal variances) or one-way ANOVA when comparing more than two groups. Differences between groups were considered to be significant if the p-value was less than 0.05.

## Results

### T-025 exhibits a potent anti-proliferative effect, particularly in highly proliferative TNBC cell lines

**Fig. 1:**
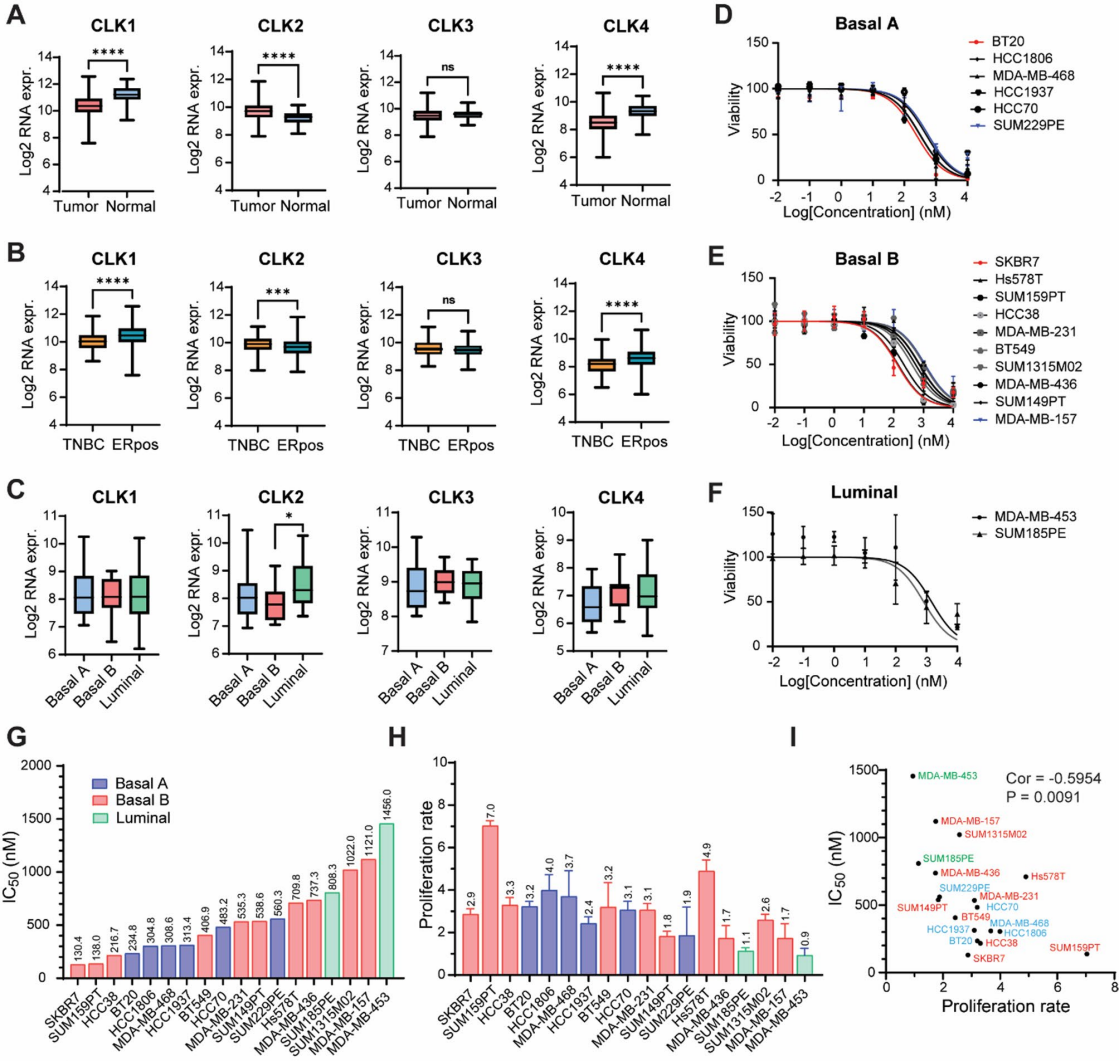
T-025 exhibits an anti-proliferative effect across a broad range of TNBC cell lines. **(A)** Log2 mRNA expression level of CLK1, CLK2, CLK3 and CLK4 in breast tumor tissue and normal tissue. **(B)** TNBC and ER-positive tumors using RNA sequencing data derived from TCGA, and **(C)** basal A, basal B and luminal breast cancer cell lines using published RNA sequencing resources [41]. *, *p* < 0.05; **, *p* < 0.01; ***, *p* < 0.001; ****, *p* < 0.0001. **(D)** Cell viability upon T-025 treatment for 72 h analyzed by the SRB assay in six basal A TNBC cell lines: BT20, HCC1806, MDA-MB-468, HCC1937, HCC70 and SUM229PE, **(E)** ten basal B TNBC cell lines: SKBR7, Hs578T, SUM159PT, HCC38, MDA-MB-231, BT549, SUM1315M02, MDA-MB-436, SUM149PT and MDA-MB-157, and **(F)** two luminal TNBC cell lines MDA-MB-453 and SUM185PE. The mean ± SD of three biological replicates is shown. 0.1% DMSO was used as a negative control. **(G)** IC_50_ of T-025 in eighteen TNBC cell lines calculated in GraphPad Prism, ranked from low to high. **(H)** 72-hour proliferation rate of eighteen TNBC cell lines using SRB absorption data of cells treated with 0.1% DMSO. **(I)** Correlation between IC_50_ of T-025 and proliferation rate in eighteen TNBC cell lines

As the CLK family is the primary target of T-025, we first analyzed the mRNA expression levels of CLK1, CLK2, CLK3 and CLK4 using RNA sequencing data derived from The Cancer Genome Atlas (TCGA) using the January 2017 version of TCGA Assembler R package. We made comparisons between breast tumor and normal tissue, TNBC and ER-positive BC tissue, and different subtypes of breast cancer cell lines using our previously published resource [41]. No significant difference was observed in CLK3 mRNA expression levels among all the comparisons (Fig. 1A-C). Interestingly, CLK1 and CLK4 mRNA expression levels were significantly lower in breast tumor-to-normal and TNBC-to-ER positive comparisons (Fig. 1A-B). Conversely, CLK2 was significantly overexpressed in breast tumor, TNBC, and luminal BC subtypes, which aligns with its role as an oncogenic kinase in breast cancer [23]. To determine the in-vitro anti-cancer effect of T-025 in TNBC, we performed a cell viability assay using the SRB assay in eighteen TNBC cell lines, which included six basal A cell lines, ten basal B cell lines and two luminal cell lines (Fig. 1D-F). T-025 exhibited an anti-cancer effect with IC_50_ values ranging from 130.4 to 1456.0 nmol/l across the eighteen TNBC cell lines (Fig. 1G). Meanwhile, we also determined cell proliferation rates using SRB absorption data of cells treated with 0.1% DMSO after 72 h (Fig. 1H). Intriguingly, we observed a significant negative correlation between IC_50_ and cell proliferation rate, with a Pearson correlation coefficient of -0.5954, indicating that T-025 preferentially targets highly proliferative TNBC cells (Fig. 1I).

### T-025 inhibits TNBC cell migration in a dose-dependent manner

**Fig. 2:**
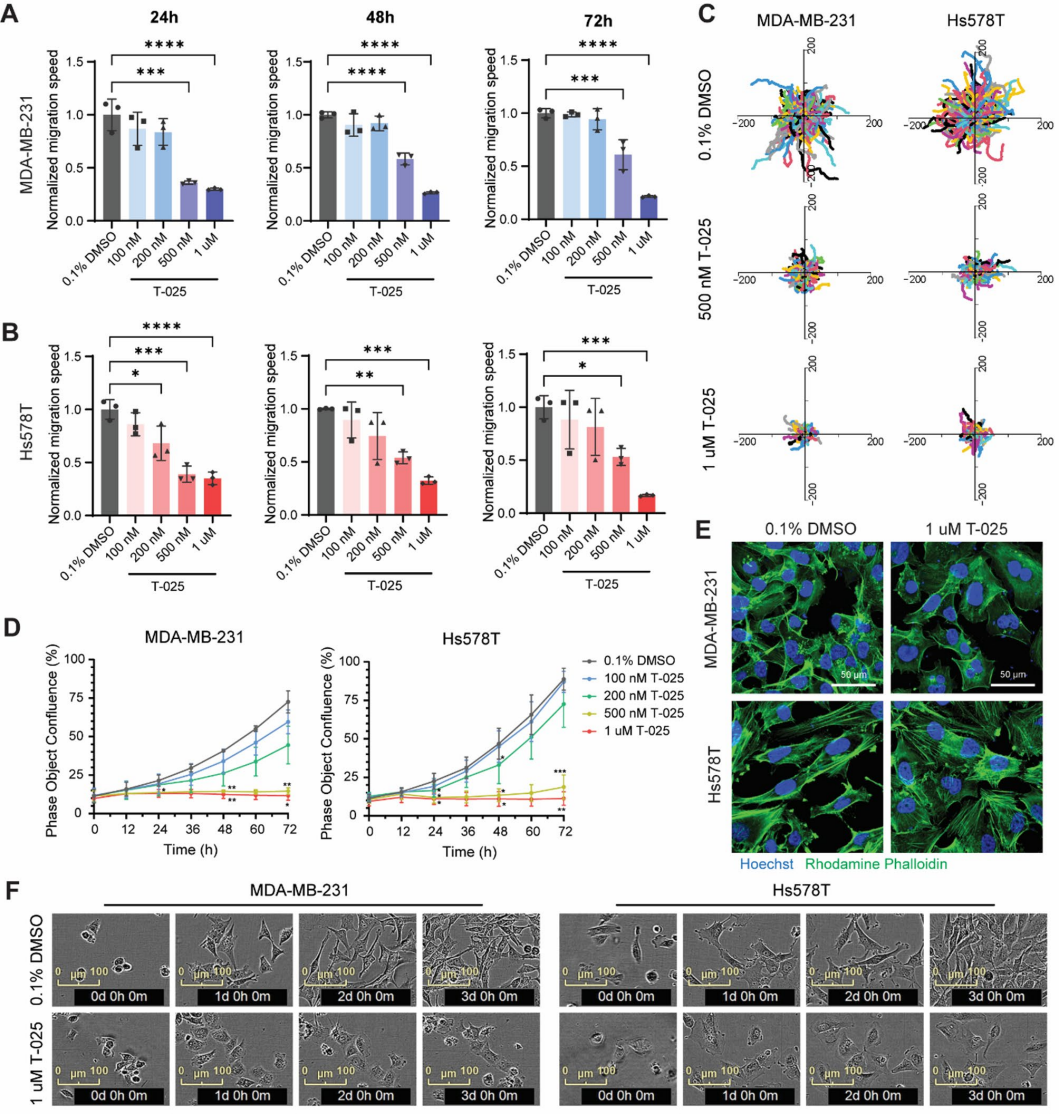
T-025 inhibits cell migration in MDA-MB-231 and Hs578T cell lines. **(A)** Normalized cell migration speed of MDA-MB-231 and **(B)** Hs578T cell lines treated with 100, 200, 500 and 1000 nM T-025 for 24, 48 and 72 h. 0.1% DMSO was used as a negative control. **(C)** Single-cell trajectories were used to visualize cell migration upon 500 nM and 1 μM T-025 treatment for 24 h in MDA-MB-231 and Hs578T cell lines. **(D)** 72-hour proliferation curves of MDA-MB-231 and Hs578T cell line treated with 100, 200, 500 and 1000 nM T-025. *, *p* < 0.05; **, *p* < 0.01; ***, *p* < 0.001; ****, *p* < 0.0001. **(E)** Confocal imaging of F-actin staining using rhodamine phalloidin in MDA-MB-231 and Hs578T cell line treated with 1 μM T-025 for 24 h was used to inspect cell morphology changes (scale bar represents 50 μm). (**F**) Brightfield images captured with the IncuCyte live cell imaging system over 72 h, showcasing MDA-MB-231 and Hs578t cells that were treated with 0.1% DMSO and 1 µM T-025. Scale bar represents 100 μm

To determine the effect of T-025 on TNBC cell migration, we performed a live-cell migration assay in two highly migratory TNBC cell lines, MDA-MB-231 and Hs578T. As shown in Fig. 2A-B, there was a clear dose-dependent effect of T-025 in migratory inhibition in both cell lines. Cell migration speed was significantly decreased upon 500 nM and 1 µM T-025 treatment after 24, 48 and 72 h in both cell lines, with 40–70% lower migration speed compared to cells with 0.1% DMSO treatment (Fig. 2A-B). Time-lapse imaging showed that MDA-MB-231 cells marked by nuclei staining actively migrated following 0.1% DMSO treatment, whereas treatment with 500 nM or 1 µM T-025 for 24 h significantly impaired cell mobility (Suppl. Movie 1–3). Additionally, the ratio of nuclei count between the final and initial timepoints, used to assess cell proliferation over 15 h, revealed a slight difference upon T-025 treatment (Suppl. Figure 1 A-B). The single cell trajectories upon 500 nM and 1 µM T-025 treatment for 24 h in both cell lines are shown in Fig. 2C. In parallel, we also monitored the proliferation curve of these two cell lines upon T-025 treatment using the IncuCyte live cell imaging system, to ascertain whether the anti-migratory effect is due to proliferation inhibition. A significant proliferation inhibition upon different concentrations of T-025 treatment at different time points was observed in both cell lines (Fig. 2D), suggesting that we cannot exclude the anti-proliferative role of T-025 in cell migration inhibition. However, we did observe a clear cell morphology difference using F-actin staining upon 1 µM T-025 treatment for 24 h in both cell lines, with cell shape changing from elongated and stretched to rounded and flat (Fig. 2E). Images captured by IncuCyte over 72 h also showed the cell morphology changes in both cell lines treated with 1 μM T-025 (Fig. 2F).

Given that T-025 exhibits significant inhibitory activity against all CLK family members, individual CLK and combination of CLK1/2/3/4 knockdown were performed to determine the contribution of CLK depletion to the anti-migratory effect of T-025. As shown in Suppl. Figure 2 A-B, CLK1, CLK2 and CLK4 knockdown slightly reduced cell migration, but CLK3 knockdown and combination knockdown of CLK1/2/3/4 significantly inhibited MDA-MB-231 cell migration, with around 25% reduction of migration speed compared with control. The single cell trajectories upon individual CLK or combination of CLK1/2/3/4 knockdown were shown in Suppl. Figure 2 C. The nuclei count from the live-cell imaging at the initial timepoint showed no difference among groups, indicating the observed reduction in cell migration was not due to variations in cell number (Suppl. Figure 2D). Furthermore, the ratio of nuclei count between the final and initial timepoints which used to assess cell proliferation over 15 h, revealed no difference upon CLKs knockdown (Suppl. Figure 2E). We next investigated whether the anti-migratory effect observed in MDA-MB-231 cells upon CLK3 knockdown was associated with its expression level. Among the four CLK family members, CLK3 exhibits high mRNA expression level across 52 breast cancer cell lines and its expression is also the highest in MDA-MB-231 cell line. (Suppl. Figure 2 F).

### T-025 induces cell cycle arrest in G1-S transition resulting in nuclei containing 4 N DNA content

**Fig. 3:**
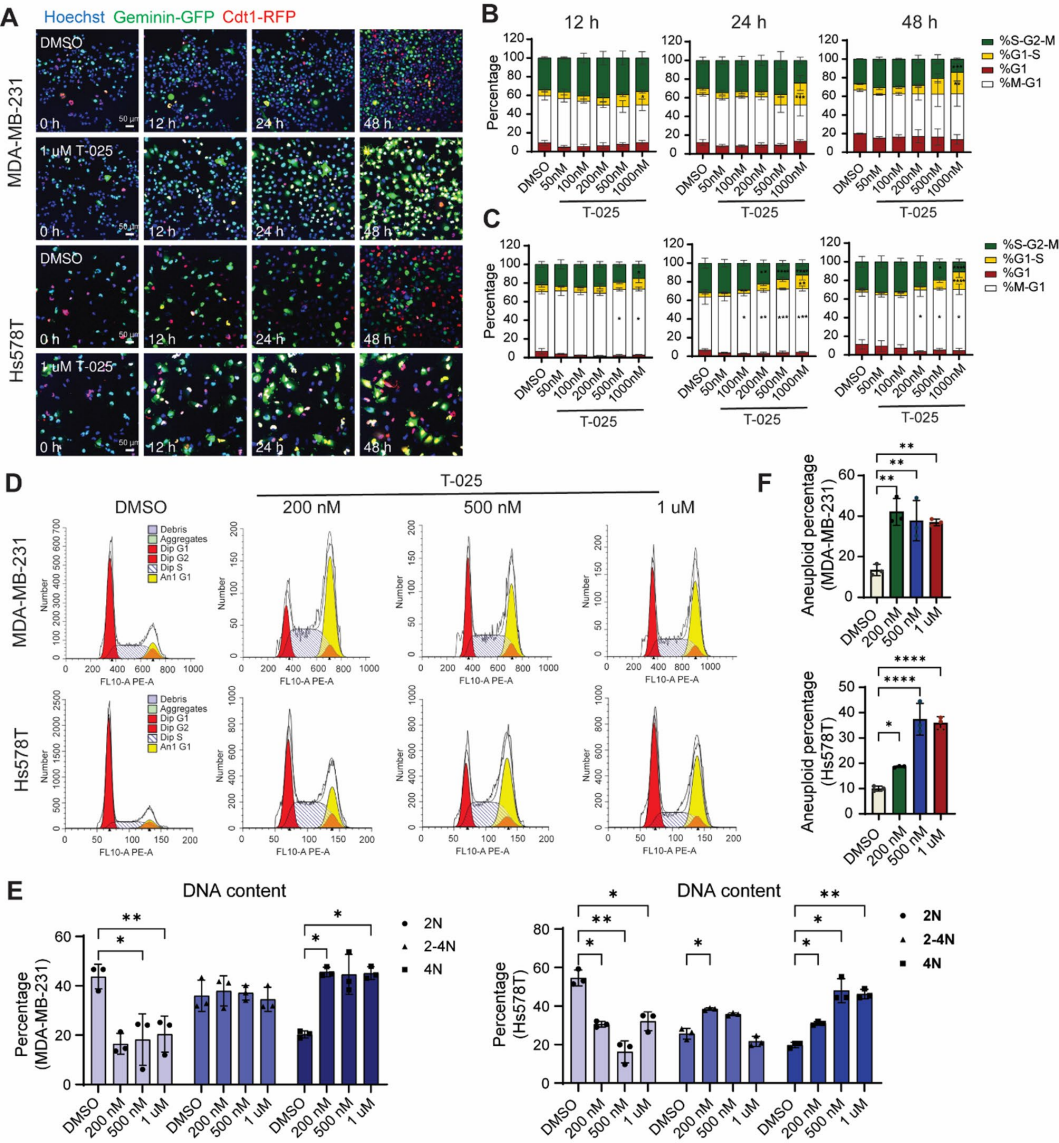
T-025 impairs the cell cycle G1-S transition, increasing the population of cells containing 4 N DNA. **(A)** Representative confocal images show MDA-MB-231 and Hs578T FUCCI reporter cells treated with 1 µM T-025 for 0, 12, 24 and 48 h. Cell cycle phases were marked by red (G1), yellow (G1-S transition), green (S-G2-M) and blue nuclei (stained by Hoechst; M-G1). **(B)** Quantifications of cell cycle phases in MDA-MB-231 and **(C)** Hs578T treated with 50, 100, 200, 500 and 1000 nM T-025 for 24 and 48 h. The mean ± SD of three biological replicates is shown. 0.1% DMSO was used as a negative control. **(D)** Flow cytometry shows the distribution of cells containing 2 N, 2–4 N and 4 N DNA content, as well as aneuploidy cells in MDA-MB-231 and Hs578T cells treated with 200, 500 and 1000 nM T-025 for 48 h. **(E)** Quantification of the percentage of cells containing 2 N (light blue), 2–4 N (medium blue) and 4 N DNA (dark blue) and **(F)** aneuploid population. The mean ± SD of three biological replicates is shown. 0.1% DMSO was used as a negative control. *, *p* < 0.05; **, *p* < 0.01. ***, *p* < 0.001; ****, *p* < 0.0001

To investigate whether the proliferative defect induced by T-025 was associated with cell cycle disruption, we first performed cell cycle analysis using FUCCI reporter cell lines and confocal imaging (Fig. 3A). We observed a significant increase in the fraction of cells undergoing G1-S transition, accompanied by a decrease in cells in S-G2-M phases in both MDA-MB-231 and Hs578T cell lines upon treatment with various concentrations of T-025 at 12, 24 and 48 h (Fig. 3A-C). Next, we performed PI staining followed by flow cytometry to determine the DNA content, and remarkably, we found a significantly higher percentage of cells containing 4 N DNA and a lower percentage of cells containing 2 N DNA in both cell lines when treated with 200 nM, 500 nM and 1 µM T-025 (Fig. 3D-E). Notably, T-025 treatment also induced an increase of the percentage of aneuploidy cells population (Fig. 3F). Taken together, these findings suggest that T-025 may affect cell proliferation by disrupting chromosomes segregation during mitosis.

### T-025 modulates alternative splicing of multiple genes related to RNA splicing, cell division and migration

**Fig. 4:**
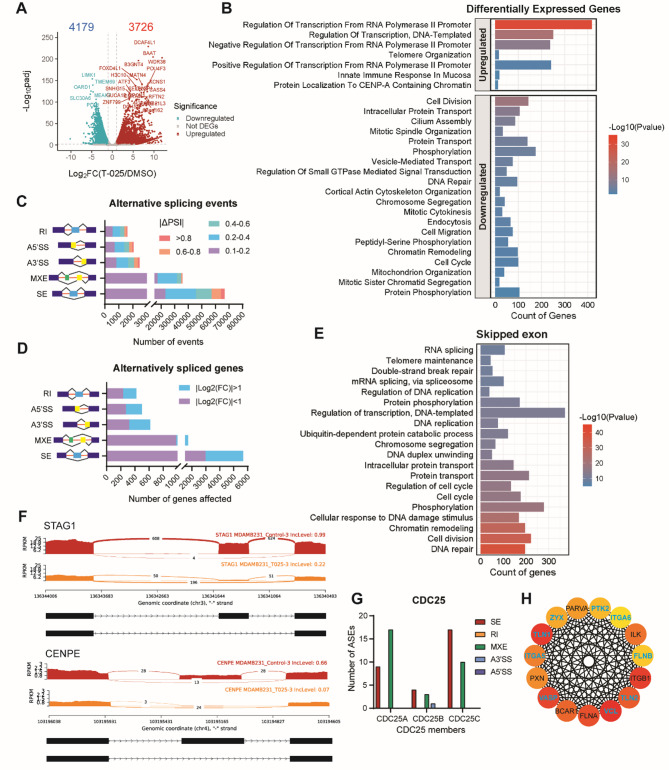
T-025 modulates alternative splicing of multiple genes related to RNA splicing, cell division and migration. **(A)** Volcano plot reveals significantly downregulated and upregulated genes in MDA-MB-231 cells treated with 1 µM T-025 for 24 h. 0.1% DMSO was used as a negative control. Red points represent upregulated genes with log_2_FC > 1.0 and an adjusted p-value of < 0.01. Blue points represent downregulated genes with log_2_FC < 1.0 and an adjusted p-value of < 0.01. Grey points represent genes with no significant difference. **(B)** GO annotation analysis shows the top 20 biological processes which enriched in the significantly upregulated and downregulated genes. **(C)** The number of different ASEs affected by T-025. The number of ASEs with average count > 10,|ΔPSI| >0.1 and FDR < 0.01 were counted and categorized by AS type and|ΔPSI|. **(D)** The number of ASGs affected by T-025 in the MDA-MB-231 cells. Genes with or without a significant difference in mRNA expression level based on DESeq2 analysis were labelled as blue and purple respectively. **(E)** GO annotation analysis shows the top 20 biological processes enriched in the ASGs affected by exon skipping with|ΔPSI| >0.4 and FDR < 0.01. **(F)** Example of Sashimi plot to visualize the alternatively spliced exon of STAG1 and CENPE (middle exon was alternatively spliced). The genomic reads densities (measured in RPKM) and junction reads (plotted as arcs) in each condition are shown. **(G)** Number of alternative spliced events of CDC25 family members CDC25A, B and C with|ΔPSI| >0.1 and FDR < 0.01. **(H)** The top 15 most important hub genes of the PPI network of focal adhesion-related genes was identified by CytoHubba in Cytoscape based on MCC algorithm. Significantly downregulated genes were labeled as blue

To get further insight into the differentially expressed genes (DEGs) and alternative splicing events (ASEs) modulated by T-025, we conducted next-generation RNA sequencing with a sequencing depth of 100 million paired reads per sample. First, we identified the DEGs in MDA-MB-231 cells upon treatment with 1 µM T-025 for 24 h. We found that 4179 genes were significantly downregulated and 3726 genes were significantly upregulated (Fig. 4A). Gene Ontology (GO) enrichment analysis suggested that the upregulated genes were strongly enriched in the regulation of transcription from the RNA polymerase II promoter processes (Fig. 4B). The downregulated genes were primarily involved in biological processes such as cell division, mitotic spindle organization and chromosome segregation. This involvement explains the observed formation of aneuploidy and cells containing 4 N DNA content (Fig. 4B).

In addition, T-025 treatment led to the downregulation of pathways associated with cell migration. These include focal adhesion (e.g. VCL, PTK2, PTK2B and RAC3), the laminin complex (e.g. LAMA5 and LAMC), extracellular matrix (ECM)-receptor interaction (e.g. ITGB3, ITGB4 and ITGB1BP1), and vascular endothelial growth factor (VEGF) signaling (e.g. KDR, PLCG1 and PTK2) (Fig. 4B, Suppl. Figure 3 A). The top 10 most significant hubs in the PPIs involved in the cell migration biological process were displayed, with KDR, PLCG1 and PTK2 ranking the highest (Suppl. Figure 3B). GO enrichment analysis in terms of cellular components revealed that 140 genes resided in focal adhesion and were strongly interconnected (Suppl. Figure 3 C-D). Interestingly, we observed a significant decrease in the expression level of genes involved in cilium assembly (Fig. 4B), such as components of intraflagellar transport (IFT) complexes and kinesin II family, which play an essential role in primary cilia formation [42].

Next, we analyzed the ASEs affected by T-025. Surprisingly, we identified more than 100,000 ASEs (|ΔPSI|>0.1, FDR < 0.01), including skipped exon (SE), mutually exclusive exon (MXE), alternative 3’ splice site (A3’SS), alternative 5’ splice site (A5’SS) and retained intron (RI), in MDA-MB-231 cells treated with 1 µM T-025 for 24 h. Among these skipped exon was the most, with around 66,000 different events (Fig. 4C). We found that exon skipping affected 5858 genes (|ΔPSI|>0.4, FDR < 0.01), among which mRNA expression level of 2698 genes was significantly altered (Fig. 4D). GO annotation analysis revealed that the genes affected by exon skipping were enriched in biological processes involved in DNA repair, protein phosphorylation and many cell division related terms (Fig. 4E). The PSI difference of splicing events of cell division related genes affected by SE was shown in Suppl. Figure 4 A. For example, stromal Antigen 1 and 2 (STAG1/2) and centromere protein E (CENPE) play a vital role in chromosome alignment and segregation [43–45]. Their alternatively spliced exons are visualized in the Sashimi plot shown in Fig. 4F. Additionally, we also discovered multiple alternative splicing events of CDC25 family members CDC25A, B and C, which are key regulators of all cell cycle stages and are required for cellular progression through G1-S transition and entry into mitosis (Fig. 4G) [46]. 163 genes affected by skipped exon upon T-025 treatment were also found to be enriched in focal adhesion, even though this term was not ranked at the top. The PSI difference of splicing events of focal adhesion-related genes affected by SE was shown in Suppl. Figure 4B. We analyzed the PPI network of these genes and identified the top 15 hub genes, including VCL, TLN1, ITGB1, VASP, FLNA, TLN2, BCAR1, ILK, ITGA5, PXN, ZYX, PARVA, PTK2, ITGA6, FLNB, most of which were downregulated as mentioned above (Fig. 4H).

Furthermore, we compared the function of ASGs affected by different alternative splicing types. Intriguingly, we found the ASGs affected by RI and A5’SS resided in the spliceosome complex, nuclear speckles and ribonucleoprotein complex, which were enriched in RNA splicing and mRNA processing pathways. In contrast, the majority of ASGs affected by SE and MXE resided in the centrosome, kinetochore, chromosome and others, which were related to cell division (Suppl. Figure 5 A-E).

### SRSF7 interactions shift with T-025: proteomics analysis reveals nuclear speckle protein and RNA machinery associations

**Fig. 5:**
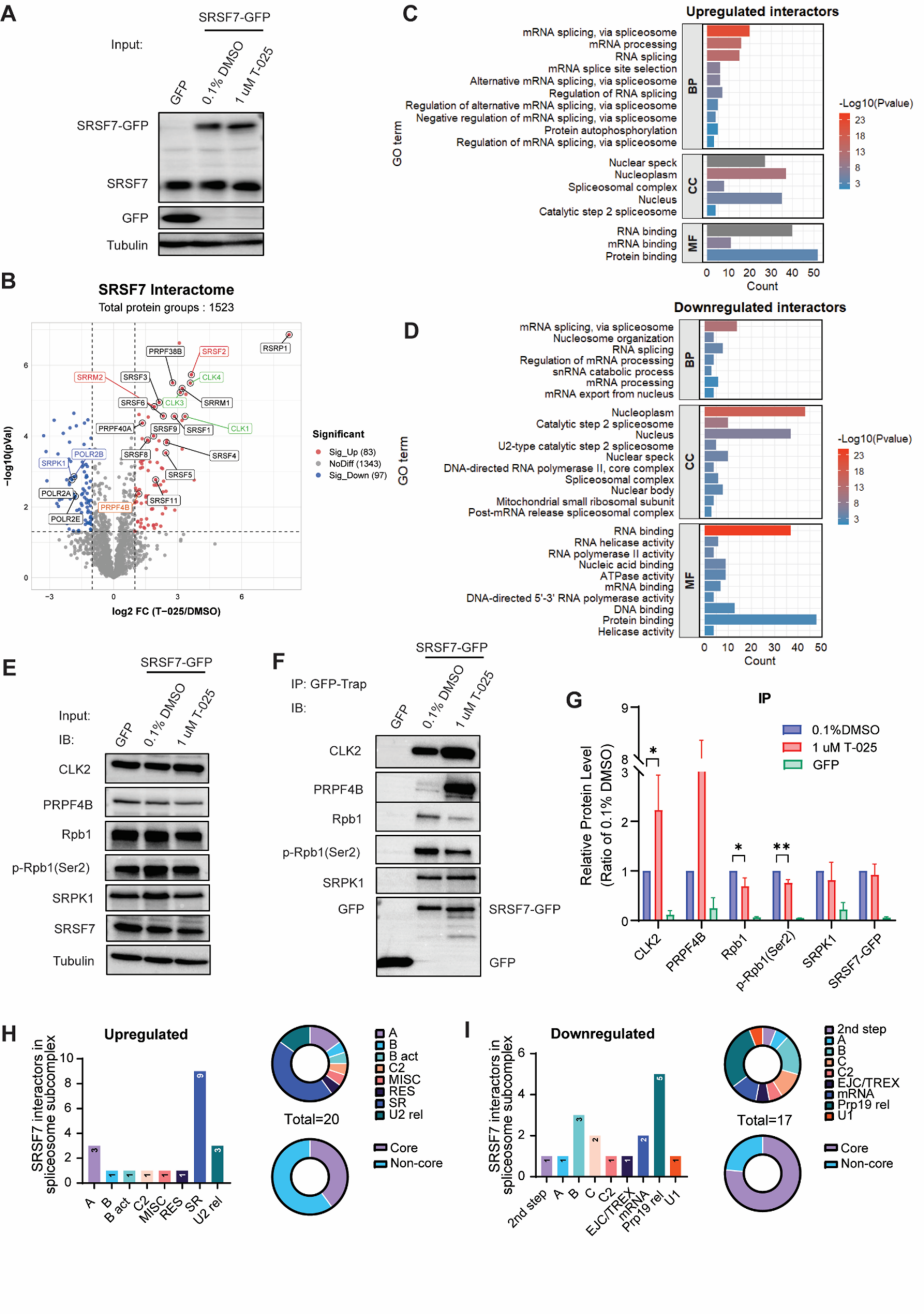
T-025 treatment alters SRSF7 interactions with nuclear speckles proteins and RNA machinery. **(A)** Western blot (WB) analysis of input samples for GFP pulldown/MS in MDA-MB-231 GFP and SRSF7-GFP expressing cells upon 1 µM T-025 treatment for 24 h. Tubulin served as the loading control. **(B)** GFP pulldown/MS analysis shows significantly upregulated and downregulated proteins in the SRSF7 interactome in MDA-MB-231 SRSF7-GFP expressing cells post 1 µM T-025 treatment for 24 h (|Log2FC|>1, FDR < 0.05). 0.1% DMSO was used as a negative control. Red points represent upregulated SRSF7 interactors with log2FC > 1.0 and an FDR of < 0.05. Blue points represent downregulated SRSF7 interactors with log2FC < 1.0 and an FDR of < 0.05. Gray points represent SRSF7 interactors with no significant difference. Experiments were performed in four independent biological replicates. **(C)** Gene ontology (GO) annotation analysis shows the biological process, cellular component, and molecular function of the upregulated and **(D)** downregulated proteins in the SRSF7 interactome. The top 10 terms with significant enrichment (FDR < 0.05) are shown. **(E)** WB of the input extracts from MDA-MB-231 GFP and SRSF7-GFP expressing cells post 1 µM T-025 treatment for 24 h. Tubulin was used as the loading control. **(F)** GFP pulldown/WB demonstrates the SRSF7 interactors of interest using GFP-Trap agarose beads. **(G)** Quantification of the expression level of SRSF7 interactors of interest in GFP pulldown/WB. Mean + SD of three independent biological replicates. Significance was determined using the Student T-test. *, *p* < 0.05; **, *p* < 0.01. **(H)** Distribution of upregulated and **(I)** downregulated SRSF7 interactors in MDA-MB-231 cells across different spliceosomal subcomplexes

SRSF7, a member of the SR protein family, plays a pivotal role in spliceosome assembly and we recently identified its importance in TNBC progression controlling (unpublished) [17]. Having observed numerous genes affected by alternative splicing upon T-025 treatment, we next investigate the impact of T-025 on SRSF7’s protein-protein interactions (PPIs), which is required for its spliceosome recruitment activity. We utilized GFP pulldown/mass spectrometry (MS) in established bacteria artificial chromosome (BAC) green fluorescent protein (GFP) MDA-MB-231 cell line that expressed SRSF7-GFP fusion product under the control of the endogenous promoter. Both endogenous SRSF7 and SRSF7-GFP fusion products were expressed in BAC-MDA-MB-231-SRSF7-GFP cells treated with 0.1% DMSO or 1 µM T-025. In contrast, the MDA-MB-231-GFP cell line, used as an experimental control in GFP pulldown, only expressed endogenous SRSF7 (Fig. 5A). As anticipated, SRSF7 interacted with a multitude of proteins, and while the total number of SRSF7 interactors remained unchanged between cells treated with 0.1% DMSO and 1 µM T-025, the composition of the SRSF7 interactome varied (Suppl. Figure 6 A-B).

Upon T-025 treatment, 55 proteins in the SRSF7 interactome were significantly upregulated, while 58 proteins were significantly downregulated (Fig. 5B, Suppl. Figure 6 A-B**)**. GO enrichment analysis revealed that both upregulated and downregulated proteins in the SRSF7 interactome were predominantly involved in RNA splicing and exhibited RNA-binding activity (Fig. 5C-D). Notably, downregulated proteins were also enriched in mRNA export from the nucleus (e.g. ALYREF, FYTTD1, CHTOP and NUP160), and spliceosomal complex assembly (e.g. SCAF11, CRNKL1 and SRPK1), underscoring the function of SRSF7 in mRNA export and spliceosome recruitment. GO cellular component analysis indicated that T-025 treatment enhanced the interaction between SRSF7 and proteins (e.g. SRRM2 and SRSF2) located in nuclear speckles, which are considered sites for the storage and modifications of splicing factors (Fig. 5C) [47]. Interestingly, GO molecular function analysis revealed an increased interaction of SRSF7 with proteins exhibiting kinase activity upon T-025 treatment including members of the CLK family (CLK1, CLK2, CLK3 and CLK4) and PRPFs family (e.g. PRPF4B, PRPF40A) (Fig. 5C, Suppl. Figure 6 C). This interaction was further validated using GFP-pull down followed by Western blotting for CLK2 and PRPF4B, with T-025 inducing a significant increase in their interaction with SRSF7, without altering their total protein level (Fig. 5E-G, Suppl. Figure 6D). Moreover, T-025 treatment resulted in a loss of interaction between SRSF7 and several proteins with RNA helicase activity (e.g. CHTOP, AQR, CHD2) and RNA polymerase activity (e.g. POLR1A, POLR2B, POLR2C and POLR2E)(Fig. 5D, Suppl. Figure 6 C). As expected, a decreased in the interaction of SRSF7 with Rpb1 and phosphorylated Rpb1 was observed, with no change in their total protein levels (Fig. 5E-G, Suppl. Figure 6D).

Since we observed a significant presence of splicing factors in the SRSF7 interactome, regardless of the treatment applied, we further explored the roles of these splicing factors within spliceosome subcomplexes based on the classification of spliceosome-associated proteins [48]. Remarkably, we discovered that 76% of the downregulated splicing factors in the SRSF7 interactome were classified as core components of the spliceosome. They were predominantly enriched in the pre-RNA processing 19 (prp19) spliceosome subcomplex (e.g. BUD31, AQR, ISY1, CRNKL1 and SYF2) and the B complex (e.g. IK, MFAP1 and ZMAT2) (Fig. 5H). Conversely, the upregulated splicing factors in the SRSF7 interactome were primarily non-core components of the spliceosome and were highly enriched in the SR protein family(Fig. 5I).

### Enhanced interaction of SRSF7 with a spectrum of phosphorylated SR and SR-like proteins following T-025 treatment

**Fig. 6:**
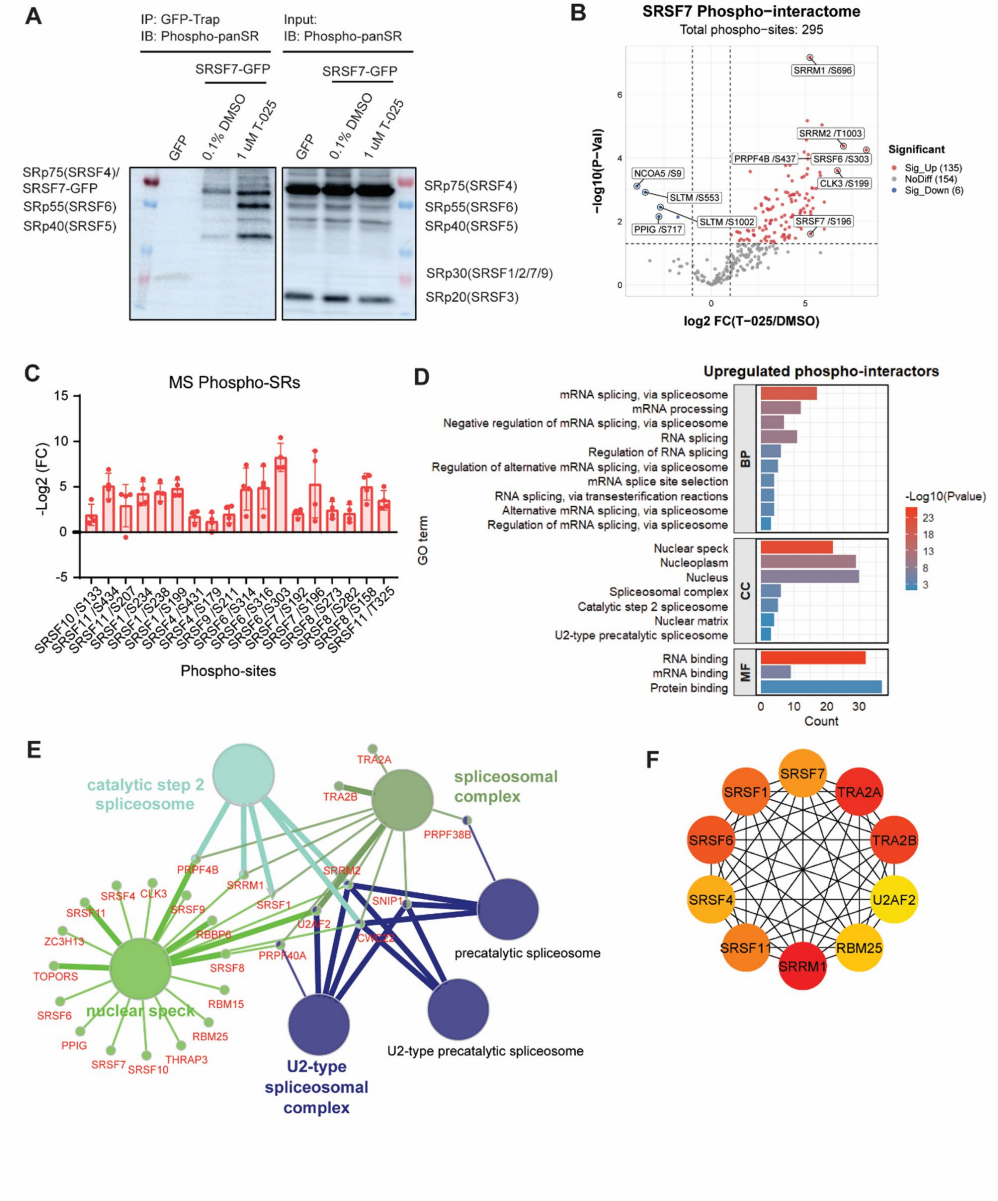
T-025 enhances the interaction of SRSF7 with a wide range of phosphorylated SR/SR-like proteins. **(A)** GFP pulldown/WB shows the interaction of phosphorylated SR proteins with SRSF7 in MDA-MB-231 cells treated with 1 µM T-025 for 24 h using anti-phospho-epitope SR proteins antibody, clone 1H4. **(B)** A volcano plot illustrates the SRSF7 phospho-interactome, as determined by GFP pulldown/MS. The experiments were performed in four independent biological replicates. Significance was determined using the Student’s T-test. Red points represent upregulated phospho-sites with log2FC > 1.0 and an FDR of < 0.05. Blue points represent downregulated phospho-sites with log2FC < 1.0 and an FDR of < 0.05. Grey points represent phospho-sites with no significant difference. **(C)** A bar graph displays the log2FC of phospho-sites from different SRSFs enriched in the SRSF7 phospho-interactome upon 1 µM T-025 treatment. **(D)** GO annotation analysis reveals the biological process, cellular component, and molecular function of the upregulated phospho-proteins in the SRSF7 phospho-interactome. The top 10 terms with significant enrichment (p- value < 0.05) are shown. **(E)** Cytoscape-ClueGo gene network interaction analysis on upregulated phospho-proteins in the SRSF7 phospho-interactome. **(F)** The top 10 most important hub genes of the protein-protein (PPI) network of upregulated phospho-proteins, identified by CytoHubba in Cytoscape based on MCC algorithm

As CLK family was demonstrated to regulate SR protein phosphorylation, we next investigated the influence of T-025 on the phosphorylation of SR proteins, which were detected with 1H4 monoclonal antibody. Immunoblotting analysis showed no significant change in the phosphorylation level of various SR proteins after a 24-hour treatment with T-025, corroborating the findings of a previous study [35]. However, our GFP pulldown/western blotting revealed an increased interaction of SRSF7 with other phosphorylated SR proteins. The specific SR proteins involved could not be precisely identified due to the low specificity of 1H4 monoclonal antibody, which detects multiple phosphorylated SR proteins (Fig. 6A). To elucidate this, we looked at the phosphorylated peptides within the SRSF7 interactome from the same GFP pulldown/MS experiment mentioned above. We found an increase in 135 phospho-sites from 42 proteins in the SRSF7 phospho-interactome, while only 6 phospho-sites from 4 proteins (PPIG, SLTM, NCOA5 and TRA2A) decreased upon T-025 treatment for 24 h (Fig. 6B). Corresponding to previous results, we observed a significant enrichment of phosphorylated SR proteins in the SRSF7 phospho-interactome upon T-025 treatment. This included phosphorylated SRSF1, SRSF4, SRSF6, SRSF7, SRSF8, SRSF9, SRSF10 and SRSF11, with phosphorylated SRSF6 being the most abundant (Fig. 6C).

GO enrichment analysis indicated the upregulated phospho-proteins were mostly involved in RNA splicing and resided in nuclear speckles with RNA binding activity. These included various SR proteins, SRRM1, SRRM2, PRPF4B, U2AF2, etc. (Fig. 6D-E). Additionally, PPIs analysis identified the top 10 hub proteins which were highly connected, including SRRM1, TRA2A, TRA2B, SRSF1, SRSF4, SRSF6, SRSF7, SRSF11, U2AF2 and RBM25. Among these, TRA2A, TRA2B and SRRM1 were the most important hubs (Fig. 6F). Altogether, our results indicated that T-025 treatment for 24 h increased the interaction of SRSF7 with a broad range of phosphorylated SR and SR-like proteins, most of which resided in nuclear speckles.

### T-025 induces accumulation of SRSF7-GFP at nuclear speckles

**Fig. 7:**
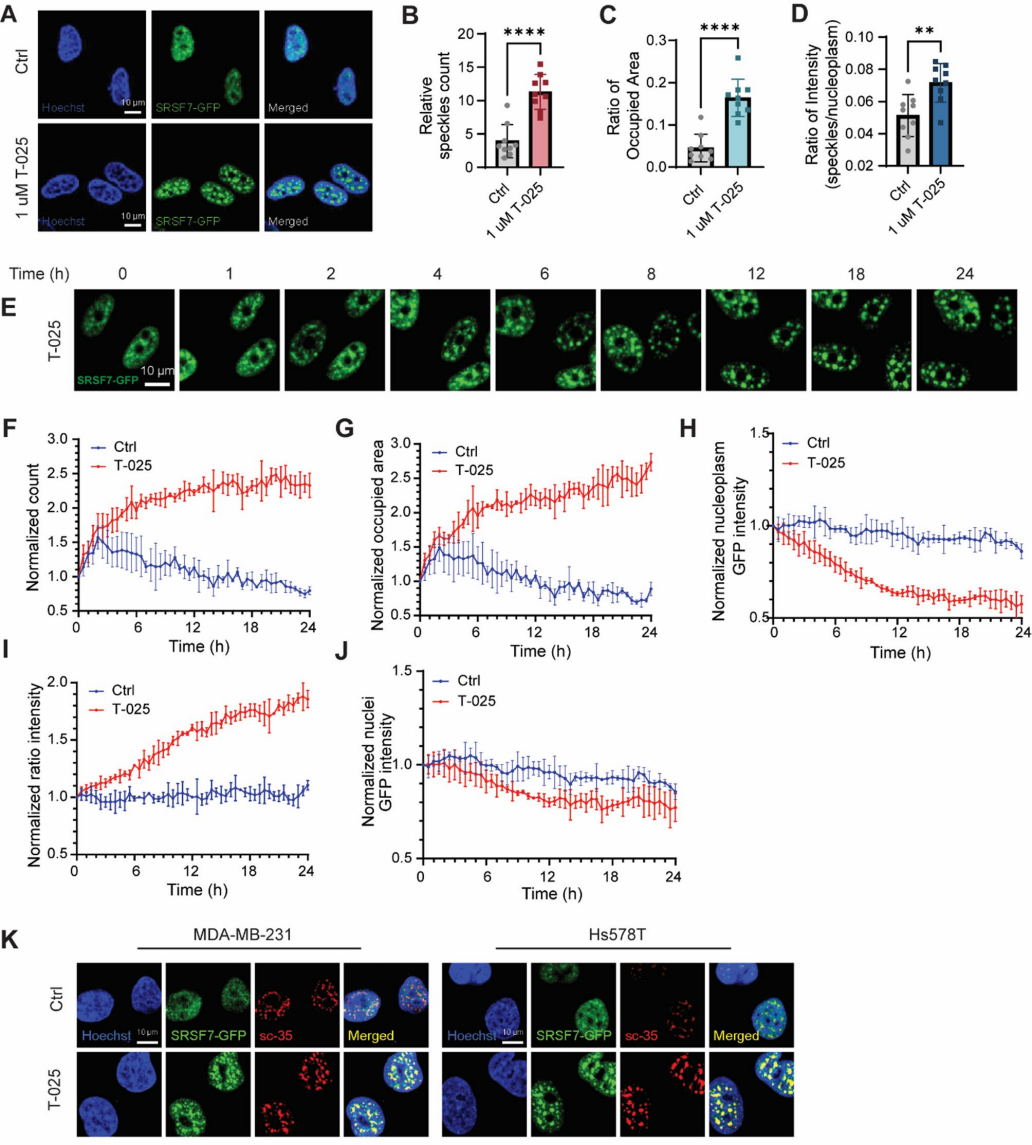
T-025 induces accumulation of SRSF7-GFP at nuclear speckles over a 24-hour period. **(A)** Confocal images show the subnuclear localization of SRSF7-GFP in Hs578T BAC-SRSF7-GFP reporter cells treated with 1 µM T-025 for 24 h. 0.1% DMSO was used as a negative control. **(B)** Quantification of counts and **(C)** occupied area of accumulated SRSF7-GFP in speckles, and **(D)** the ratio of GFP integrated intensity between speckles and the nucleoplasm. Error bars represent the measurements from 9 images captured per group. **, *p* < 0.01; ****, *p* < 0.0001. **(E)** Representative time-lapse confocal images show the SRSF7-GFP accumulation in speckles in Hs578T BAC-SRSF7-GFP reporter cells treated with 1 µM T-025 in 24 h. **(F)** Quantifications of counts and **(G)** occupied area of accumulated SRSF7-GFP in speckles, **(H)** nucleoplasm GFP intensity, **(I)** the ratio of GFP integrated intensity between speckles and the nucleoplasm and **(J)** the intensity of total GFP in nuclei. All measurements were normalized to the first imaging time point. **(K)** Immunofluorescence microscopy images show the localization of SRSF7-GFP and the nuclear speckles marker, SC-35 in MDA-MB-231 and Hs578T BAC-SRSF7-GFP reporter cells treated with 1 µM T-025 for 24 h

Given the observation of that SRSF7 interacts more with the nuclear speckles-resided proteins, we next imaged the subnuclear localization of SRSF7 proteins in cells treated with T-025 for 24 h. Using an established BAC-GFP Hs578T cell line that expressed SRSF7-GFP fusion product, we found an altered distribution of SRSF7-GFP fusion protein in the nuclei, changing from a homogeneous diffuse expression to speckles-like localization (Fig. 7A). Image quantifications showed a significant increase in the counts and occupied area of speckles, and the ratio of integrated intensity between speckles and the nucleoplasm after 1 µM T-025 treatment for 24 h (Suppl. Figure 7A, Fig. 7B-D). Additionally, we also observed a similar accumulation of other splicing factors, SNRPD2 and SNRPD3, in the BAC-Hs578T-SNRPD2-GFP and BAC-Hs578T-SNRPD3-GFP reporter cell lines respectively upon T-025 treatment for 24 h (Suppl. Figure 7B-D).

Next, to further explore the dynamic accumulation of SRSF7 induced by T-025, we performed time-lapse live cell imaging using BAC-Hs578T-SRSF7-GFP cell line over 24 h. Representative time-lapse images of cells treated with T-025 for 24 h are shown in Fig. 7E. Similarly, we quantified the images using the established analysis pipeline. As shown in Fig. 7F-G, SRSF7 accumulation in the speckle-like structures appeared as early as 1 h after T-025 treatment. The SRSF7-GFP intensity in the nucleoplasm was continuously decreased over 24 h after T-025 treatment (Fig. 7H). The counts and occupied area of speckles, and the ratio of integrated intensity between speckles and nucleoplasm were constantly increased over time (Fig. 7F-G, I). However, we also measured the total GFP intensity measured in the entire nuclei and found no significant difference when comparing T-025 treated cells with control cells, suggesting that the observed SRSF7 accumulation was not due to an increase in its protein level (Fig. 7J). Of note, the measured slight decrease in nuclear SRSF7-GFP fluorescence intensity was considered to be the consequence of photobleaching during imaging.

To confirm that the structures where SRSF7 accumulates are nuclear speckles, we performed immunostaining using a nuclear speckle marker SC-35 (also called SRSF2) and indeed found a significant co-localization between SRSF7-GFP and SC-35 staining in both MDA-MB-231 and Hs578T cell lines upon 1 µM T-025 treatment for 24 h, which was in line with our previous findings. Additionally, we also observed that the nuclear speckles were enlarged in size in both cell lines with T-025 treatment, indicating aberrant RNA splicing was taking place (Fig. 7K).

### T-025 reduces the motility of SRSF7-GFP protein accumulated at nuclear speckles

**Fig. 8:**
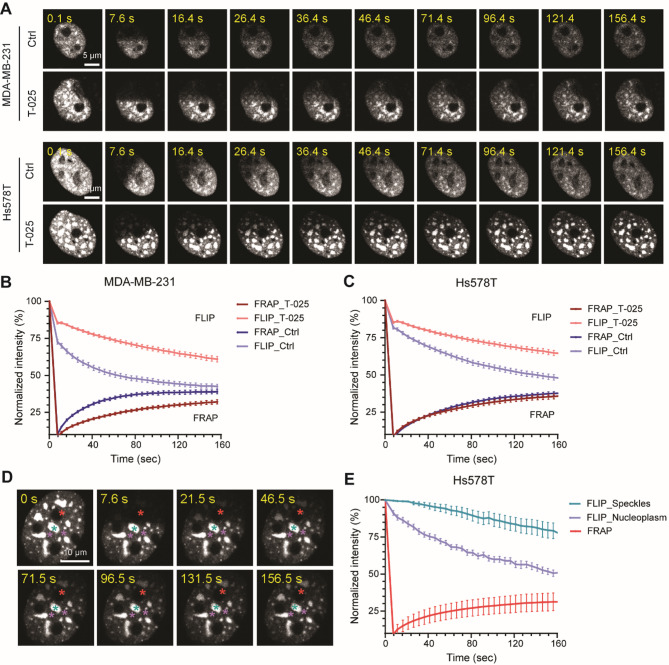
T-025 reduces the dynamic movement of SRSF7-GFP accumulated at nuclear speckles. **(A)** Representative time-lapse confocal images display frames from a photobleaching experiment in MDA-MB-231 and Hs578T BAC-SRSF7-GFP reporter cells treated with 1 µM T-025 for 24 h. 0.1% DMSO was used as a negative control. **(B)** The graph shows the normalized fluorescence intensity of SRSF7-GFP in the unbleached and bleached areas of MDA-MB-231 and **(C)** Hs578T BAC-SRSF7-GFP reporter cells treated with 1 µM T-025 for 24 h. Bars represent the mean ± SEM, *n* = 16–23 cells for each condition. **(D)** Representative frames from a photobleaching experiment in Hs578T SRSF7-GFP expressing cells with 1 µM T-025 treatment for 24 h are shown. The half-bleached area is labelled with a red asterisk, and the nuclear speckle and nearby nucleoplasm area were labelled with green and purple asterisks, respectively, in the images. **(E)** The graph shows the normalized fluorescence intensity of SRSF7-GFP in the bleached area (red), nuclear speckle (green) and nearby nucleoplasm area (purple) in Hs578T SRSF7-GFP expressing cells treated with 1 µM T-025 for 24 h. Bars represent the mean ± SEM, *n* = 11 cells for each condition

Given the accumulation of SRSF7 at nuclear speckles upon T-025 treatment, we further investigated the effect of T-025 on the dynamic mobility of SRSF7. We performed fluorescence recovery after photobleaching (FRAP) and fluorescence loss in photobleaching (FLIP) using BAC-Hs578T-SRSF7-GFP and BAC-MDA-MB-231-SRSF7-GFP reporter cell lines treated with 1 µM T-025. Following photobleaching of SRSF7-GFP in one-half of the nuclei, the fluorescence recovery in the bleached area and the loss of fluorescence in the unbleached area were measured every 5 s over 2.5 min (Fig. 8A). The SRSF7-GFP intensity quickly recovered in the bleached area and was lost fast in the unbleached area similarly in both MDA-MB-231 and Hs578T cell lines treated with 0.1% DMSO, finally equilibrating to a more homogeneous distribution (Fig. 8B-C). In contrast, in cells treated with 1 µM T-025 for 24 h, the SRSF7-GFP intensity recovered and was lost much slower in the bleached and unbleached areas respectively in MDA-MB-231 cell line (Fig. 8B). Interestingly, we only observed a slower fluorescence loss in the unbleached area but no difference of fluorescence recovery in the bleached area in the Hs578T cell line treated with 1 µM T-025 for 24 h compared to cells treated with 0.1% DMSO (Fig. 8C).

Having observed more and larger speckles where SRSF7 resided in the Hs578T cell line with T-025 treatment, we hypothesized that the recovery of SRSF7-GFP in the bleached area was preferentially and mostly coming from the SRSF7-GFP in the nucleoplasm of unbleached area. To demonstrate this, following photobleaching of SRSF7-GFP in one-half of the nuclei, we not only measured the fluorescence recovery in the bleached area but also the loss of fluorescence at SRSF7 accumulated sites (speckles) and the nearby nucleoplasm, instead of the entire unbleached area (Fig. 8D). Indeed, the normalized intensity of SRSF7-GFP decreased much more rapidly at the nucleoplasm than at the speckles where SRSF7 accumulated (Fig. 8E). Together these data suggest that T-025 could reduce the mobility of SRSF7 and halt the release of SRSF7 from nuclear speckles, thereby decreasing the efficiency of its spliceosome recruitment activity.

## Discussion

Emerging studies have discovered that dysregulation of alternative splicing plays an essential role in nearly all hallmarks of cancer, including sustained proliferation and metastasis formation [20, 36, 49, 50]. Inhibitors that target splicing factors, either directly or indirectly could modulate RNA splicing programming and have therefore emerged as a novel and effective anti-cancer therapeutic strategy in recent years [29, 35, 51–53]. Here, we evaluated the efficacy of a new and potent CLK inhibitor T-025 in two crucial cancer hallmarks: cell cycle and cell migration. We unraveled the impact of T-025 in SR protein activity and RNA splicing in TNBC cell lines. T-025 leads to the accumulation of splicing factors at nuclear speckles and stalls their release to splicing sites, resulting in the RNA splicing reprogramming of a large number of genes involved in cell division and migration. Although T-025 was also shown to inhibit DYRK1 family with similar *K*_d_ value as CLK family, the minimal effect of DYRK1 family inhibition by T-025 was suggested [35]. Additionally, our pulldown/MS results confirmed a direct interaction of SRSF7 with all the CLK family members, whereas with none of the DYRKs. Collectively, our findings highlight the important role of CLK in RNA splicing and provide evidence supporting the possibility that CLK inhibition by T-025 could be a promising therapeutic approach for TNBC patients.

It has been reported that MYC hyperactivation increases global pre-mRNA synthesis, leading to an enhanced dependency on the spliceosome. Therefore, MYC-driven tumors are more vulnerable to spliceosome inhibition [35, 54, 55]. CLK play an essential role in MYC-driven tumors, and increased expression of CLK2 reduces the prognosis of MYC-amplified breast cancer patients [35]. MYC commonly drives TNBC which is more aggressive than other breast cancer subtypes. In the present study, we demonstrated the efficacy of T-025 on cell migration in two highly migratory TNBC cell lines. RNA sequencing uncovered multiple genes, which were alternatively spliced and involved in cell migration-related pathways upon T-025 treatment, such as the Rac/PAK and focal adhesion kinase (FAK) signaling pathways. For more than 20 years it was shown that the expression of FAK is frequently upregulated in many cancers, including breast cancer, and correlated with tumor progression and metastasis [42, 56]. Paxillin (PXN) is vital to drive the assembly of focal adhesion and its disruption could reduce breast cancer cell migration [57]. Our data showed that 162 genes related to focal adhesion were alternatively spliced by exon skipping, such as PTK2, PXN and VCL, and their mRNA expression level were also downregulated. Furthermore, FAK is activated by integrins and the association of FAK and β4 integrin contributes to the malignancy of TNBC [58, 59]. We discovered the downregulation of multiple integrin family members such as ITGB4, ITGB3, ITGB1BP1 and ITGB3BP, upon T-025 treatment. The alternatively spliced transcript variants via exon skipping of ITGB1BP1 and ITGB3BP were also found. It was reported that ITGB1BP1 can promote the tumor cell migration and invasion, and ITGB3BP was associated with poor prognosis of glioma [60, 61]. However, the role of different spliced transcript variants of ITGB1BP1 and ITGB3BP in focal adhesions and tumor cell migration remains unclear. Further studies should address this question through inhibition of different ITGB1BP1 transcript variants using specific siRNAs and determine the effect of their inhibitions on malignant characteristics in-vitro. Additionally, it has been demonstrated that the loss of primary cilium can cause aberrant cell adhesion, leading to defect of spreading-dependent cell migration [42]. Our data showed a significant decrease in the expression level of genes involved in cilium assembly such as components of IFT complexes and kinesin II family, which play a pivotal role in primary cilia formation [42]. Therefore, we anticipate that CLK pharmacological inhibition by T-025 could block cell migration through modulating focal adhesions in TNBC.

Furthermore, we demonstrated the in-vitro efficacy of T-025 in a broad range of TNBC cell lines. We found that T-025 could impair the cell cycle G1 to S transition and induce the accumulation of cells containing 4 N DNA content. RNA sequencing revealed multiple alternatively spliced genes involved in cell division and chromosome segregation, located at the centriole, centrosome, kinetochore and mitotic spindle. As mentioned above, STAG1/2 are crucial subunits of the cohesin protein complex, which are required for chromosome segregation and proper DNA repair [43, 44]. CENPE, a member of the kinesin-7 family, plays a vital role in chromosome alignment [45]. The alternative splicing of CENPE was also reported in prostate cancer cells upon CLK pharmacological inhibition by TG003, where the expression of exon 38 inclusion isoform significantly increased upon TG003 treatment [53]. While in this study the accumulation of exon 38 inclusion isoform was not observed in TNBC cells upon T-025 treatment, we did find the significant exon-skipping events in exon 17/18 of CENPE (ΔPSI < -0.9). A recent study showed that RSRC2 depletion results in exon 17 skipping of CENPE, subsequently leading to chromosome congression defects, suggesting that CENPE pre-mRNA splicing plays a pivotal role in cell cycle regulation [62]. Interestingly, these cell division-related genes tend to be affected by skipped exons and mutually exclusive exons, suggesting that different genes might rely on distinct splicing modes to become functional. Notably, we discovered multiple alternative splicing events of CDC25A, B and C, accompanied by mRNA expression level downregulated of CDC25B and CDC25C. CDC25 phosphatases are key regulators of all cell cycle stages and are required for cellular progression through controlling cyclin-dependent kinase (CDK) dephosphorylation to ensure G1-S transition and entry into mitosis [46]. They can be regulated via changing of protein level and alternative splicing, while the role of different alternative splicing isoforms is not much known yet [63]. Additionally, the overexpression of CDC25A, B and C were found in many cancer types, e.g., lung cancer, breast cancer and liver cancer [64, 65]. Their overexpression was often associated with a shorter overall survival rate and poorer prognosis in patients [64, 66]. Inhibition of CDC25 using RNA interference and pharmacological inhibition arrests cancer cell proliferation both in-vitro and in-vivo [65, 67].

Nuclear speckles have been identified as storage and modification sites for splicing factors. In addition, some kinases (such as CLK and PRPF4B) and phosphatases involved in the phosphorylation/dephosphorylation of splicing factors are also localized in nuclear speckles [47]. Approximately 80%-85% of pre-mRNA splicing occurs co-transcriptionally, whereas 15–20% of splicing takes place post-transcriptionally, predominantly at nuclear speckles. Polyadenylated pre-mRNAs that have not completed splicing accumulate in nuclear speckles along with their associated spliceosomes [68]. Our data showed that CLK inhibition by T-025 led to an accumulation of SRSF7 in nuclear speckles and restricted its dynamic mobility. Furthermore, GFP pulldown followed by mass spectrometry revealed an increased interaction of SRSF7 with proteins localized in nuclear speckles, and a decreased interaction with proteins associated with RNA polymerase II and RNA helicase activity. This suggests that co-transcriptional splicing may be suppressed upon CLK inhibition, as SR proteins are crucial components of the spliceosome for the recognition of splicing sites and spliceosome assembly [69, 70]. To verify this hypothesis, the activated spliceosome should be quantified in fractionated nuclei containing nucleoplasmic fraction and chromatin-associated fraction respectively. Moreover, cross-linking immunoprecipitation (CLIP)-seq in both nucleoplasm and chromatin-associated material can be used to gain more comprehensive readout of the interactions of SRSF7 with downstream targeting pre-mRNAs undergoing co-/post-transcriptionally splicing. Additionally, we observed a higher concentration of splicing factors, particularly SR proteins, in nuclear speckles. PPIs analysis identified the top 10 hub proteins with TRA2A, TRA2B and SRRM1 being the most important hubs. The TRA2 protein, consisting of transformer 2 alpha homolog (TRA2A) and transformer 2 beta homolog (TRA2B), belongs to the serine/arginine-rich (SR) protein family and it has been shown to be dysregulated in many types of tumors and to play a crucial role in splicing regulation [71–73]. Serine/arginine repetitive matrix 1 (SRRM1) is an SR-like protein and has been considered a splicing coactivator [74]. It remains unclear whether the incomplete pre-mRNA splicing continues to generate mature mRNA in nuclear speckles. However, our deep RNA sequencing data revealed that numerous genes are affected by different types of alternative splicing, suggesting that splicing occurring in nuclear speckles may also be influenced. Further studies are needed to answer this question by sequencing the mRNAs in different subnuclear fractions. A recent study has revealed that the spatial organization of RBPS networks is essential for regulating RNA splicing, where core spliceosomal components and accessary RBPs are recruited to pre-mRNA forming distinctly distributed meshworks [75]. Our study demonstrated that CLK pharmacological inhibition by T-025 significantly changed the PPI networks of SR protein, which may lead to the disruption of the normal spatial organization of RBPs and subsequent defection of RNA splicing process.

Given that T-025 modulates pathways related to cell migration, further investigations are warranted to evaluate its efficacy in TNBC progression and metastasis in-vivo. Additionally, it remains essential to determine whether the in-vivo functionality of T-025 is in line with that observed in-vitro. To this end, proteomics approaches such as pulldown followed by MS and transcriptomic analysis via deep RNA-sequencing of primary xenograft tumors could be performed to confirm its role in the disruption of spliceosomal complex and splicing reprogramming observed in-vitro. Although minimal impact of T-025 on body weight has been reported in mice, a more comprehensive in-vivo safety assessment could be carried out, e.g. evaluation of T-025 distribution and accumulation dynamics in tumors and major organs [35].

One of the limitations of this study is that CLK inhibition by T-025 was not tested in healthy human cells, such as breast epithelial cells and fibroblasts. Having observed that T-025 preferentially targets highly proliferative TNBC cells, we could speculate that T-025 might also inhibit the proliferation of the normal highly proliferative human cells, such as activated immune cells and hematopoietic stem cells. Therefore, the potential effect of T-025 on these cell types needs to be addressed in further study. Furthermore, worthy to note is that SRSF7 is not the only substrate of CLKs. The observed alterations of phenotypes and alternative splicing are likely the accumulated consequences of disruption of multiple SR proteins, not only SRSF7, upon CLK inhibition by T-025. Additionally, CLKs were shown to phosphorylate other RS domain-containing splicing factors, such as U2AF65 (also known as U2AF2) [76]. The aberrant phosphorylation of U2AF2 was also observed in SRSF7 phospho-interactome upon T-025 treatment.

In conclusion, we have uncovered the impact of pharmacological inhibition of CLK by T-025 on the spliceosome complex and the transcriptional responses in regard to TNBC cell proliferation and migration. T-025 treatment leads to the accumulation of SR protein at nuclear speckles and restricts its dynamic mobility. This results in abnormal spliceosome recruitment and immensely aberrant RNA splicing of genes related to cell division and migration, ultimately leading to mitotic defects and impaired migration. We anticipate that targeting CLK could potentially be an effective therapeutic strategy for the development of anti-cancer drugs in TNBC.

## Supplementary Information

Below is the link to the electronic supplementary material.

Supplementary Material 1: **Fig. 1.** The ratio of nuclei count between the final and initial timepoints (t50/t01) in live-cell migration assay in (**A**) MDA-MB-231 and (**B**) Hs578T cells following T-025 treatment for 24, 48 and 72 hours. *, p < 0.05; **, p < 0.01; ***, p < 0.001; ****, p < 0.0001

Supplementary Material 2: **Fig. 2.** Effect of CLKs knockdown on TNBC cell migration. (**A**) MDA-MB-231 cell migration speed upon single CLK1, CLK2, CLK3, CLK4 and combination of CLK1/2/3/4 knockdown. siKinasePool was used as a negative siRNA control. Experiment was performed in two independent biological replicates. *, p < 0.05. (**B**) Violin plot showing the distribution of single-cell migration speed upon CLKs knockdown. Two independent biological replicates were plotted separately. (**C**) Single-cell trajectories were used to visualize the cell migratory behavior upon CLKs knockdown in MDA-MB-231 cells. (**D**) The quantification of nuclei count in MDA-MB-231 cells upon CLKs knockdown at the first timepoint of time-lapse imaging. (**E**) The ratio nuclei count of the last timepoint (t60) and first timepoint (t01). (**F**) Heatmap showing the log2 mRNA expression level of CLK1, CLK2, CLK3 and CLK4 in 52 breast cancer cell lines

Supplementary Material 3: **Fig. 3.** Analysis of deep RNA sequencing data for MDA-MB-231 cells treated with 1 µM T-025. (**A**) PPIs analysis on downregulated genes related to cell migration. (**B**) The top 10 most important hub genes of the PPIs identified by CytoHubba in Cytoscape based on MCC algorithm. (**C**) The top 20 GO cellular components terms enriched in downregulated genes. (**D**) PPI network of the downregulated genes involved in focal adhesion

Supplementary Material 4: **Fig. 4.** Heatmap of the percentage spliced‐in value difference (ΔPSI) of exon skipping events of (**A**) cell division-related genes and (**B**) focal adhesion-related genes upon T-025 treatment. An unique number was given to different SE event of an individual gene and only the SE events with|ΔPSI| greater than 0.4 were plotted in the heatmap

Supplementary Material 5: **Fig. 5.** The top 20 GO cellular component terms significantly enriched in the ASGs affected by different ASEs. The top 20 GO cellular components terms enriched in the ASGs affected by (**A**) skipped exon, (**B**) mutually exclusive exon, (**C**) alternative 5’ splice site, (**D**) retained intron and (**E**) alternative 3’ splice site with|ΔPSI| >0.4 and FDR < 0.01

Supplementary Material 6: **Fig. 6.** Identification of the SRSF7 interactome in MDA-MB-231 SRSF7-GFP expressing cells via GFP pulldown/MS. (**A**) The SRSF7 interactome in cells treated with 0.1% DMSO and (**B**) 1 µM T-025. Red points represent proteins enriched in the SRSF7 interactome with log2FC >1.0 and an FDR of < 0.05 as compared to MDA-MB-231-GFP cells. (**C**) The log2 fold change in the abundance of PRPFs, CLKs and RNA polymerase II subunits between cells treated with 1 µM T-025 and 0.1%DMSO. (**D**) Quantification of the input in GFP pulldown/Western blot

Supplementary Material 7: **Fig. 7.** (**A**) The analysis pipeline in CellProfiler software was used to measure the count and occupied area of accumulated SRSF7-GFP speckles, as well as the integrated intensity of SRSF7-GFP at speckles and in the nucleoplasm. (**B**) Representative confocal microscopy images show the accumulation of SNRPD2-GFP and SNRPD3-GFP as speckles in the nuclei of Hs578T cells upon treatment with 1 µM T-025. (**C**) Quantifications of the counts and occupied area of accumulated SNRPD2-GFP and SNRPD3-GFP speckles, and the ratio of GFP integrated intensity between speckles and nucleoplasm. All measurements were normalized to the first imaging time point. 0.1% DMSO was used as a negative control. ***, p < 0.001; ****, p < 0.0001

Supplementary Material 8

Supplementary Material 9

Supplementary Material 10

Supplementary Material 11

## Data Availability

The authors confirm that the data supporting the findings of this study are available within the article [and/or] its supplementary materials. The mass spectrometry proteomics data have been deposited to the ProteomeXchange Consortium via the PRIDE partner repository with the dataset identifier PXD058273. The RNA sequencing data have been deposited to ArrayExpress with the accession number E-MTAB-14664. The 24-hour time-lapse live-cell imaging data and photobleaching experiment data have been deposited to BioImage Archive with the accession number S-BIAD1498 and S-BIAD1501 respectively. PRIDE reviewer account details: Username: reviewer_pxd058273@ebi.ac.uk Password: dr98MbMvojrY.

## Abbreviations

A3’SS: Alternative 3’ splice site
A5’SS: Alternative 5’ splice site
AS: Alternative splicing
ASE: Alternatively spliced gene
BAC: Bacterial artificial chromosome
CLIP: Cross-linking immunoprecipitation
DEG: Differentially expressed gene
FLIP: Fluorescence loss in photobleaching
FRAP: Fluorescence recovery after photobleaching
FUCCI: Fluorescent Ubiquitination-Based Cell Cycle Indicator
GO: Gene ontology
MS: Mass spectrometry
MXE: Mutually exclusive exon
PPI: Protein-protein interaction
PSI: Percentage spliced-in value
RBP: RNA-binding protein
RCM: Random cell migration
RI: Retained intron
RRM: RNA recognition motif
SE: Skipped exon
SR: Serine and arginine-rich
TNBC: Triple-negative breast cancer
