# Supplementary figures and images for: Pharmacological CLK inhibition disrupts SR protein function and RNA splicing blocking cell growth and migration in TNBC
Source: Breast Cancer Res. 2025 Jul 29;27:140. doi: 10.1186/s13058-025-02091-w (PMC12309053; doi:10.1186/s13058-025-02091-w)

**A** MDA-MB-231

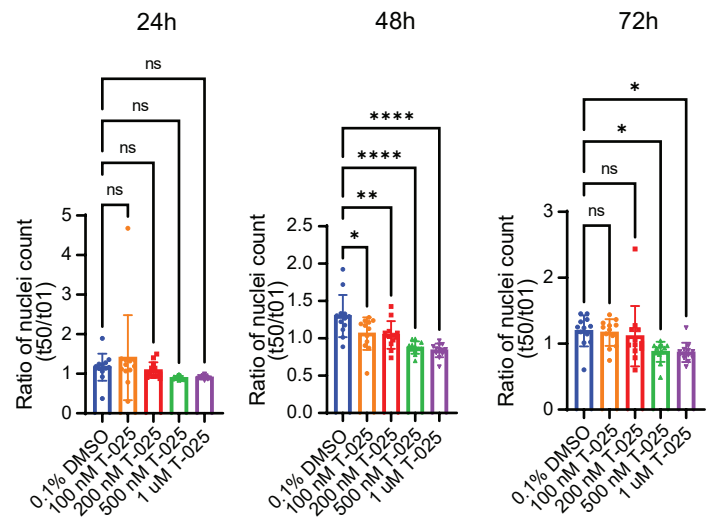

**B** Hs578T

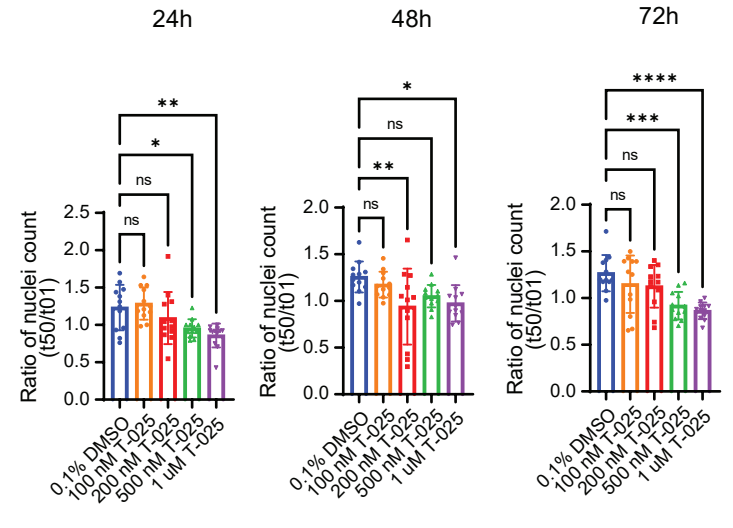

Supplement: Supplementary file 1 — Supplementary Material 1: Fig. 1. The ratio of nuclei count between the final and initial timepoints (t50/t01) in live-cell migration assay in (A) MDA-MB-231 and (B) Hs578T cells following T-025 treatment for 24, 48 and 72 hours. *, p < 0.05; **, p < 0.01; ***, p < 0.001; ****, p < 0.0001 [file 13058_2025_2091_MOESM1_ESM.pdf]

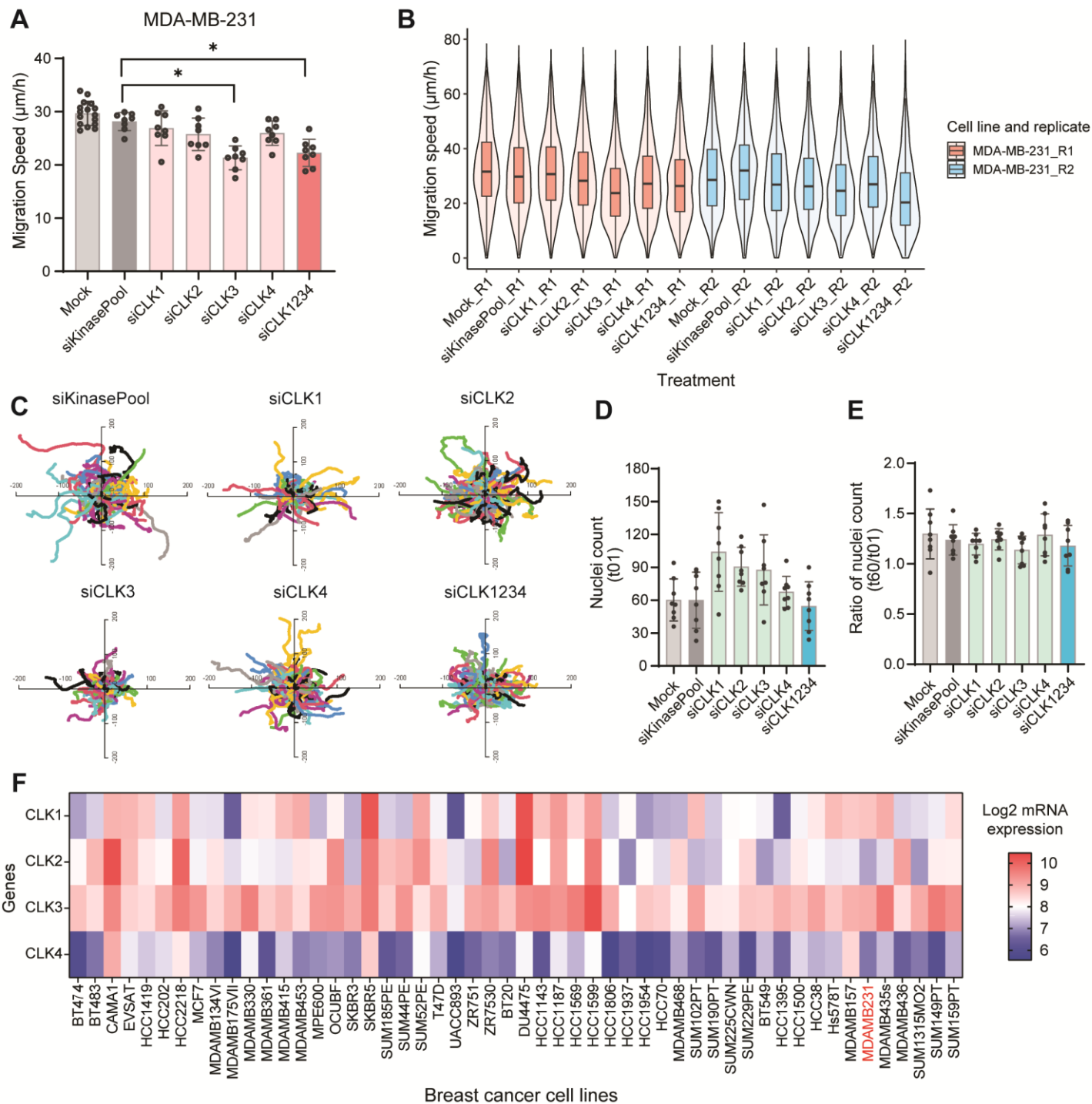

Supplement: Supplementary file 2 — Supplementary Material 2: Fig. 2. Effect of CLKs knockdown on TNBC cell migration. (A) MDA-MB-231 cell migration speed upon single CLK1, CLK2, CLK3, CLK4 and combination of CLK1/2/3/4 knockdown. siKinasePool was used as a negative siRNA control. Experiment was performed in two independent biological replicates. *, p < 0.05. (B) Violin plot showing the distribution of single-cell migration speed upon CLKs knockdown. Two independent biological replicates were plotted separately. (C) Single-cell trajectories were used to visualize the cell migratory behavior upon CLKs knockdown in MDA-MB-231 cells. (D) The quantification of nuclei count in MDA-MB-231 cells upon CLKs knockdown at the first timepoint of time-lapse imaging. (E) The ratio nuclei count of the last timepoint (t60) and first timepoint (t01). (F) Heatmap showing the log2 mRNA expression level of CLK1, CLK2, CLK3 and CLK4 in 52 breast cancer cell lines [file 13058_2025_2091_MOESM2_ESM.pdf]

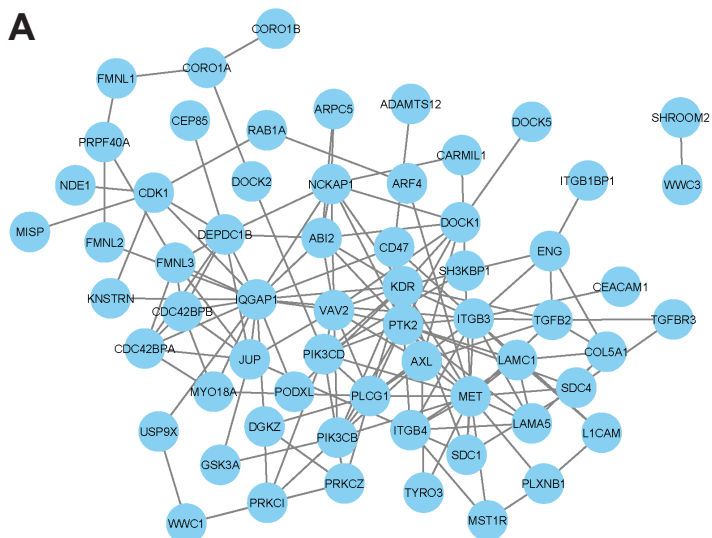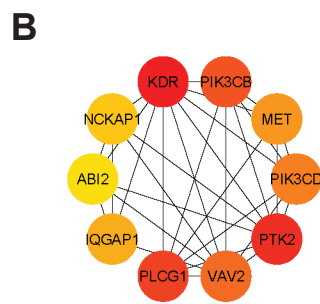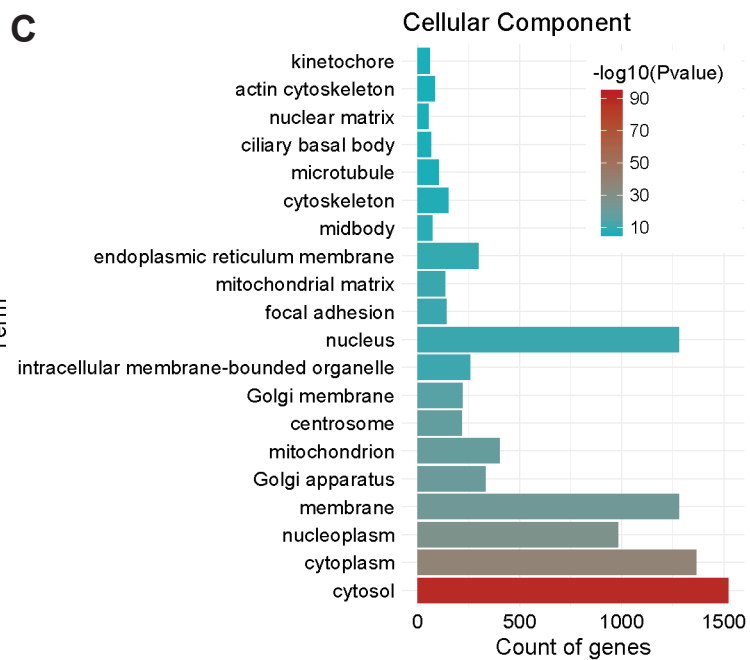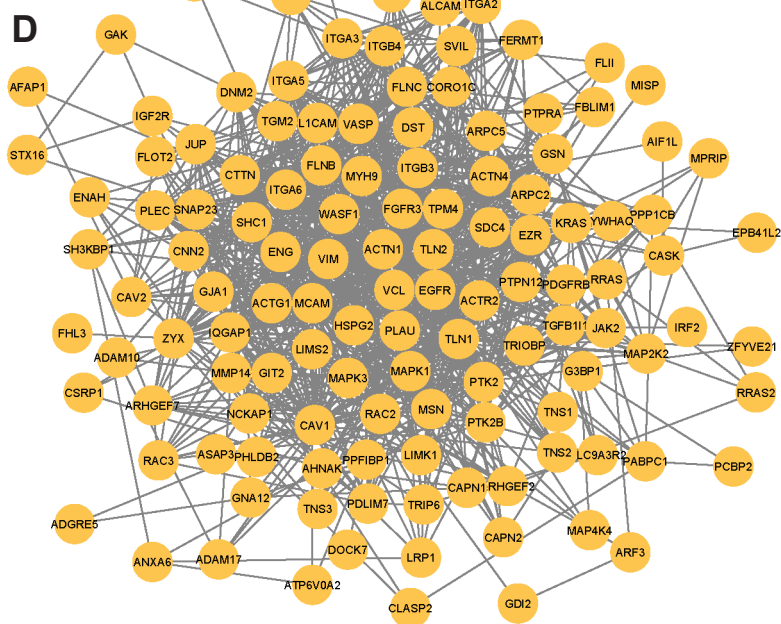

Supplement: Supplementary file 3 — Supplementary Material 3: Fig. 3. Analysis of deep RNA sequencing data for MDA-MB-231 cells treated with 1 µM T-025. (A) PPIs analysis on downregulated genes related to cell migration. (B) The top 10 most important hub genes of the PPIs identified by CytoHubba in Cytoscape based on MCC algorithm. (C) The top 20 GO cellular components terms enriched in downregulated genes. (D) PPI network of the downregulated genes involved in focal adhesion [file 13058_2025_2091_MOESM3_ESM.pdf]

**A**

GO:0051301  
Cell division related genes affected by SE

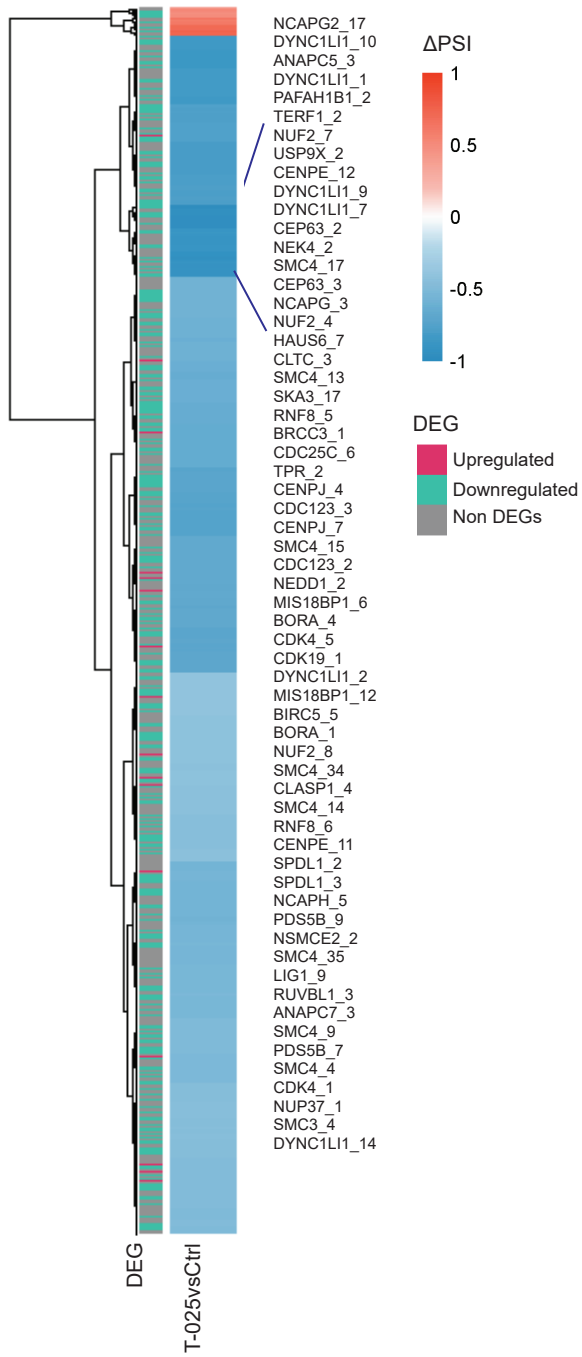**B**

GO:0005925  
Focal adhesion related genes affected by SE

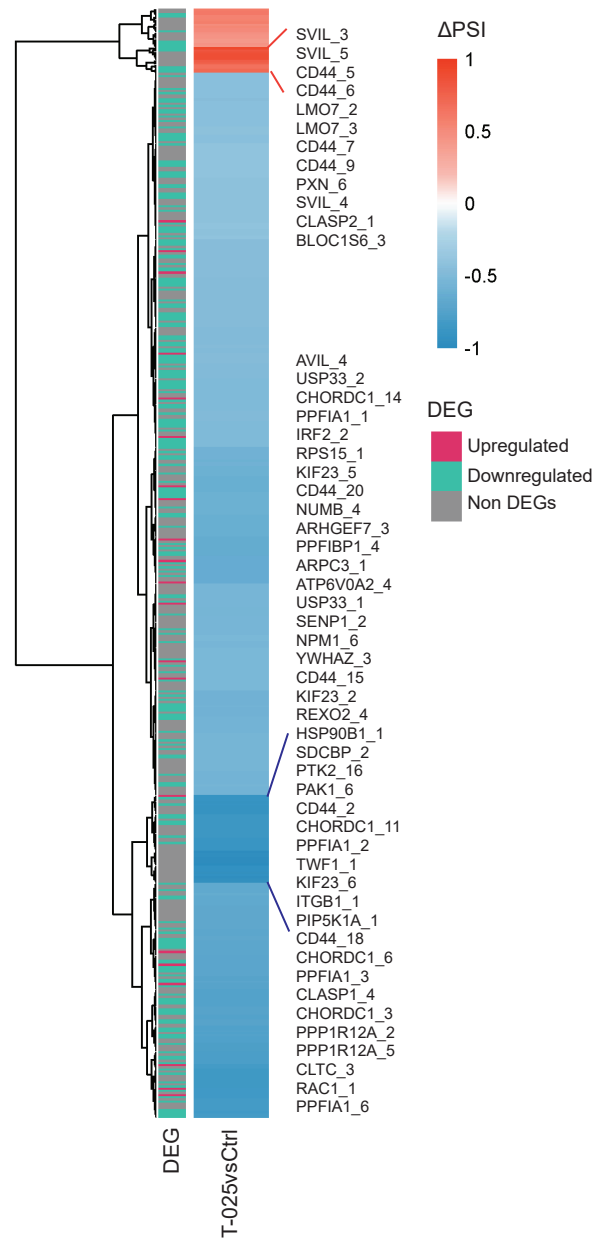

Supplement: Supplementary file 4 — Supplementary Material 4: Fig. 4. Heatmap of the percentage spliced‐in value difference (ΔPSI) of exon skipping events of (A) cell division-related genes and (B) focal adhesion-related genes upon T-025 treatment. An unique number was given to different SE event of an individual gene and only the SE events with|ΔPSI| greater than 0.4 were plotted in the heatmap [file 13058_2025_2091_MOESM4_ESM.pdf]

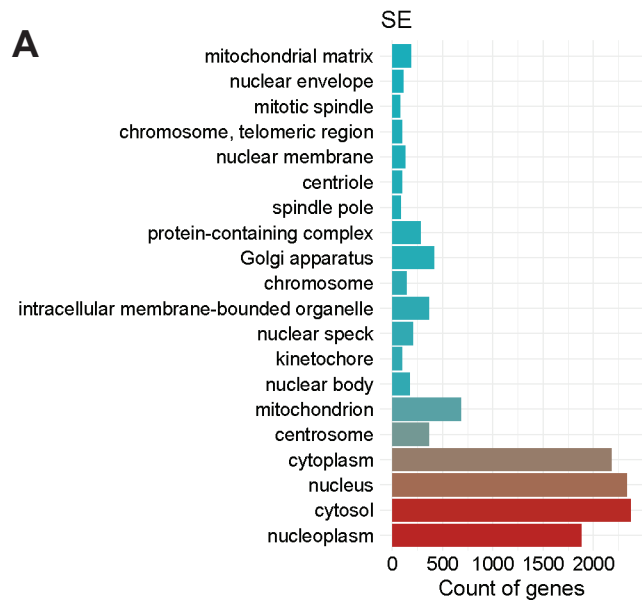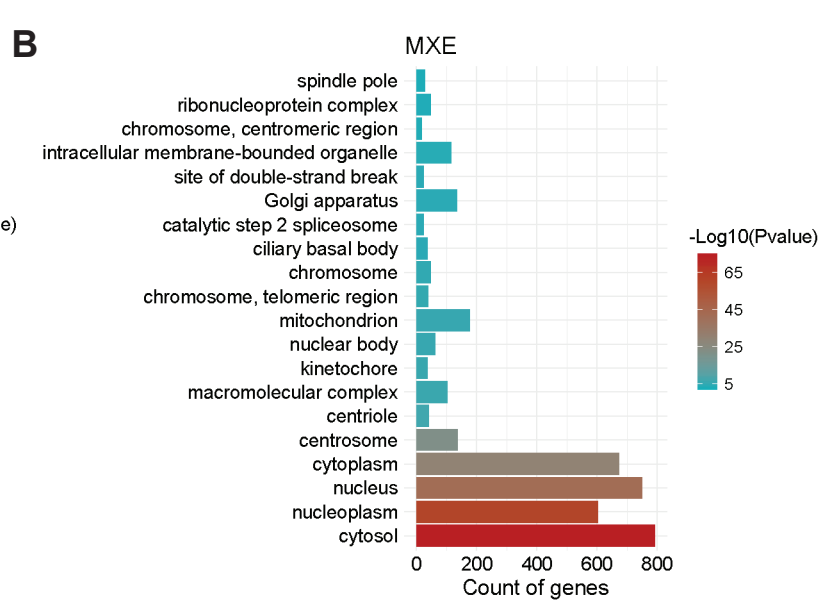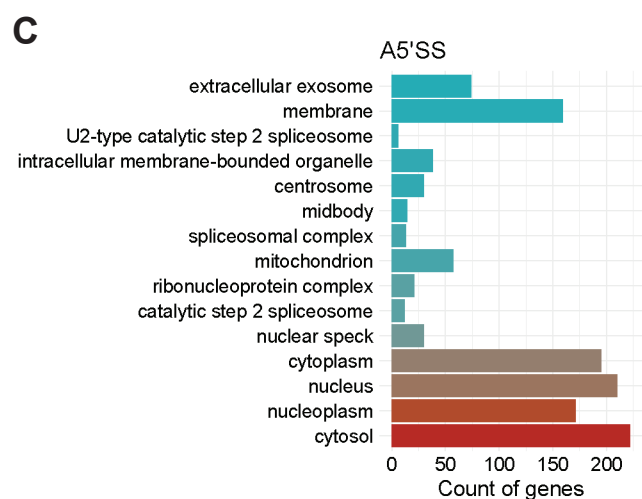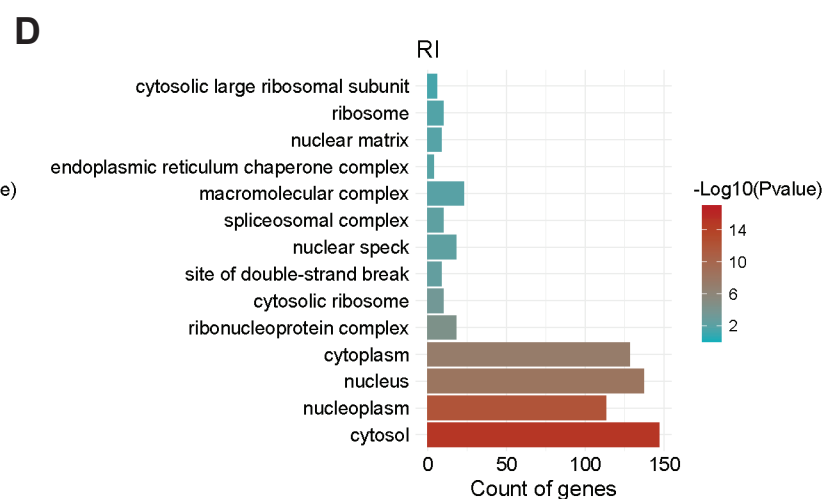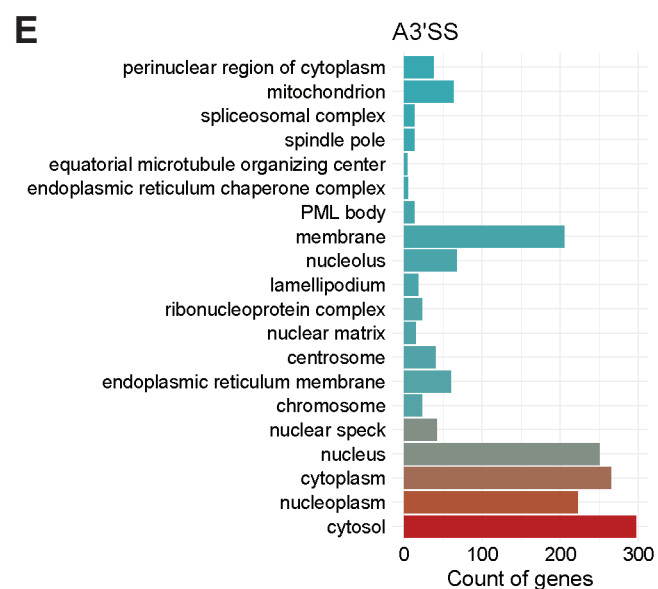

Supplement: Supplementary file 5 — Supplementary Material 5: Fig. 5. The top 20 GO cellular component terms significantly enriched in the ASGs affected by different ASEs. The top 20 GO cellular components terms enriched in the ASGs affected by (A) skipped exon, (B) mutually exclusive exon, (C) alternative 5’ splice site, (D) retained intron and (E) alternative 3’ splice site with|ΔPSI| >0.4 and FDR < 0.01 [file 13058_2025_2091_MOESM5_ESM.pdf]

## SRSF7 Interactome

Total protein groups : 1559

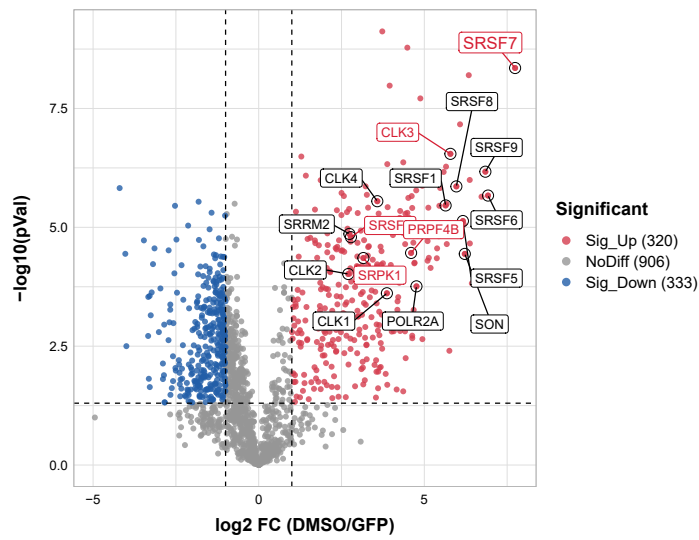

**B**

## SRSF7 Interactome

Total protein groups : 1554

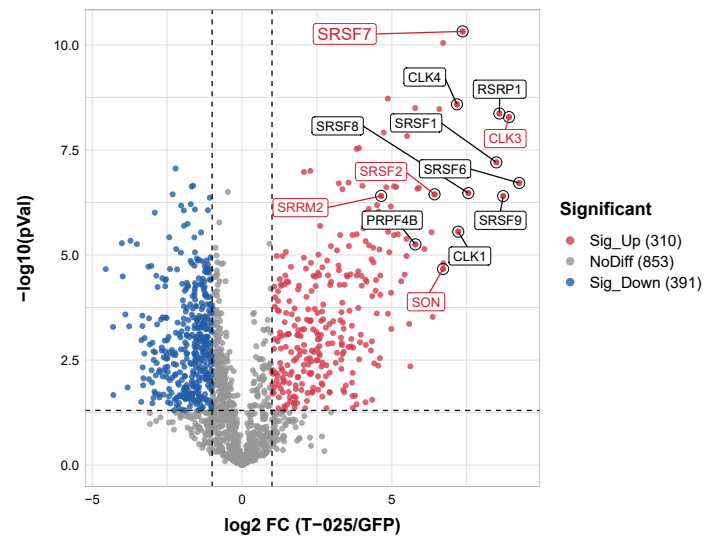

**C**

## MS

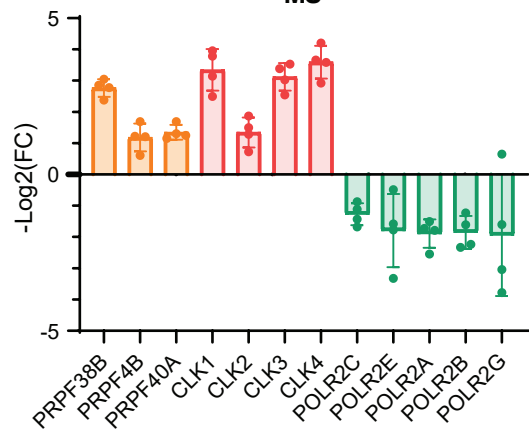

D

## Input

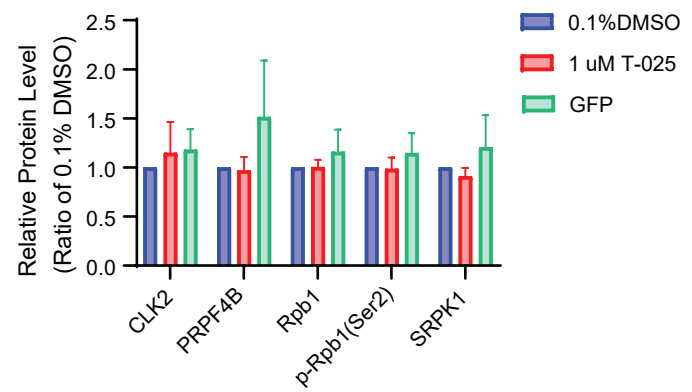

Supplement: Supplementary file 6 — Supplementary Material 6: Fig. 6. Identification of the SRSF7 interactome in MDA-MB-231 SRSF7-GFP expressing cells via GFP pulldown/MS. (A) The SRSF7 interactome in cells treated with 0.1% DMSO and (B) 1 µM T-025. Red points represent proteins enriched in the SRSF7 interactome with log2FC >1.0 and an FDR of < 0.05 as compared to MDA-MB-231-GFP cells. (C) The log2 fold change in the abundance of PRPFs, CLKs and RNA polymerase II subunits between cells treated with 1 µM T-025 and 0.1%DMSO. (D) Quantification of the input in GFP pulldown/Western blot [file 13058_2025_2091_MOESM6_ESM.pdf]

A

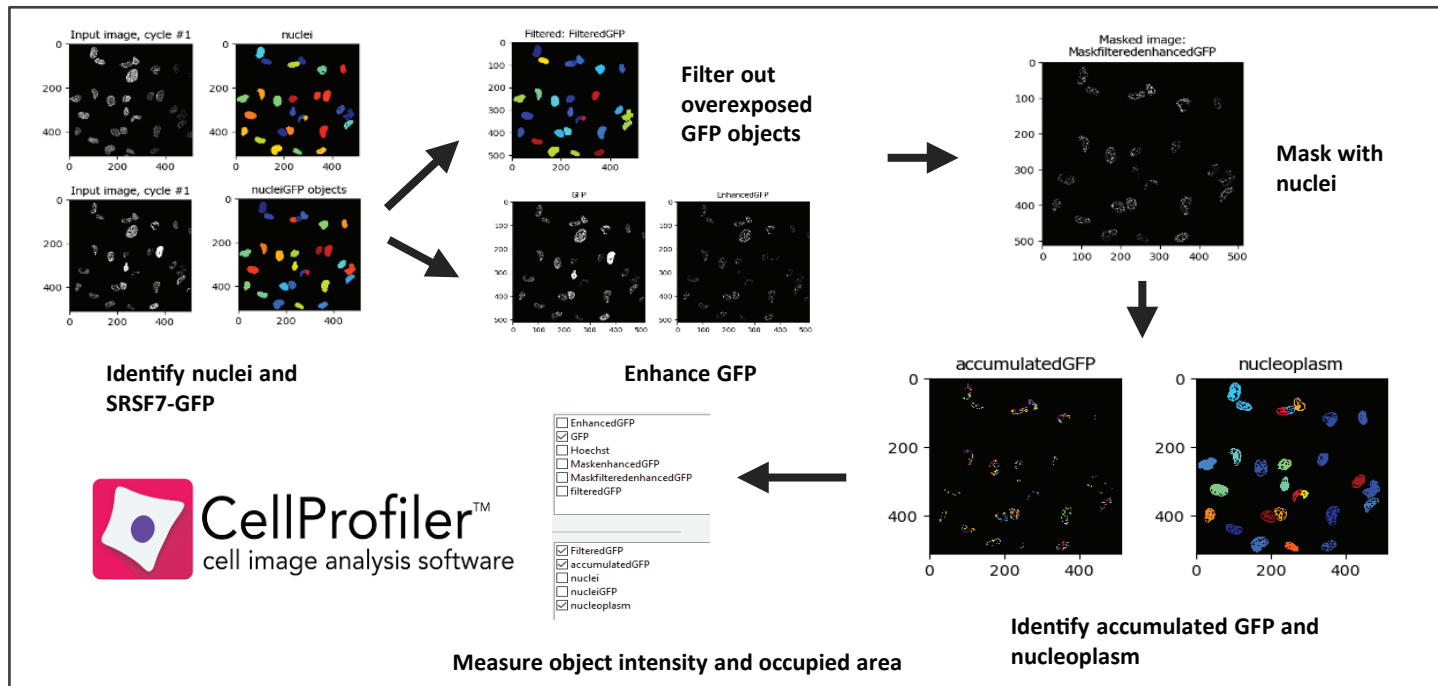

B

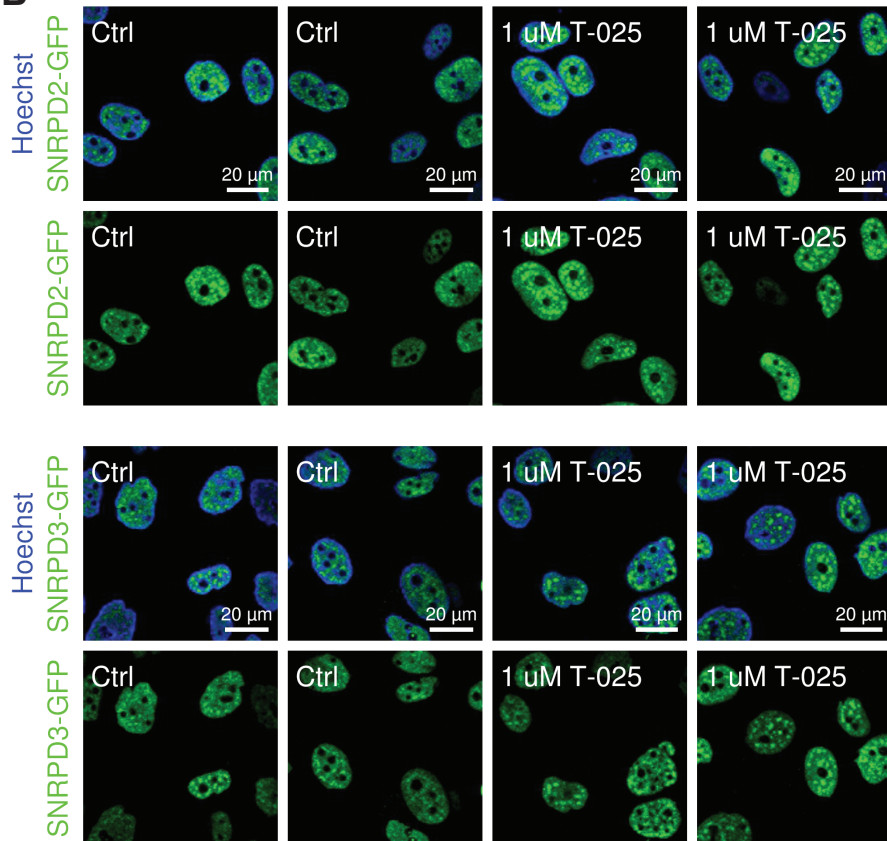

C

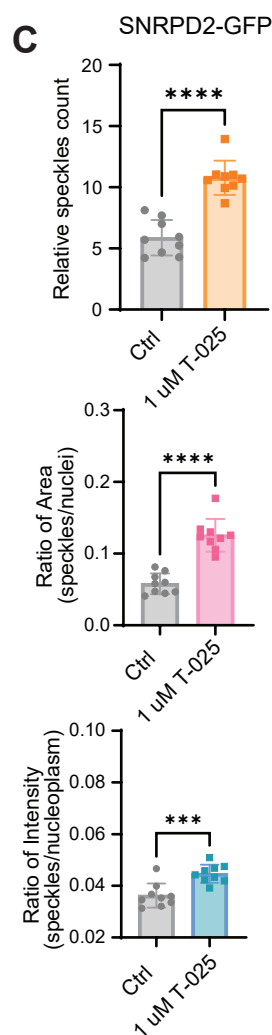

D

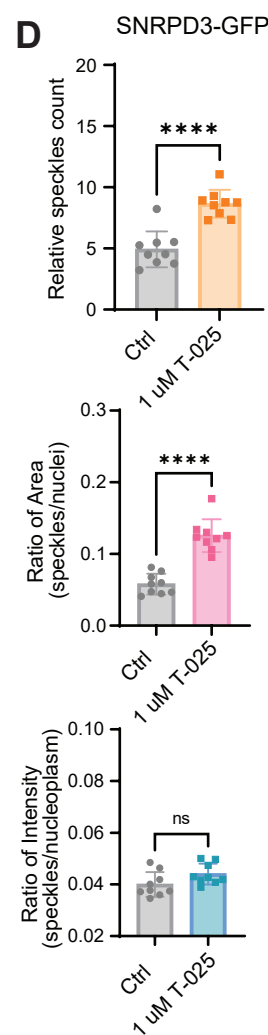

Supplement: Supplementary file 7 — Supplementary Material 7: Fig. 7. (A) The analysis pipeline in CellProfiler software was used to measure the count and occupied area of accumulated SRSF7-GFP speckles, as well as the integrated intensity of SRSF7-GFP at speckles and in the nucleoplasm. (B) Representative confocal microscopy images show the accumulation of SNRPD2-GFP and SNRPD3-GFP as speckles in the nuclei of Hs578T cells upon treatment with 1 µM T-025. (C) Quantifications of the counts and occupied area of accumulated SNRPD2-GFP and SNRPD3-GFP speckles, and the ratio of GFP integrated intensity between speckles and nucleoplasm. All measurements were normalized to the first imaging time point. 0.1% DMSO was used as a negative control. ***, p < 0.001; ****, p < 0.0001 [file 13058_2025_2091_MOESM7_ESM.pdf]
