# Supplementary material for: Pharmacological CLK inhibition disrupts SR protein function and RNA splicing blocking cell growth and migration in TNBC
Source: Breast Cancer Res. 2025 Jul 29;27:140. doi: 10.1186/s13058-025-02091-w (PMC12309053; doi:10.1186/s13058-025-02091-w)
Supplement: Supplementary file 11 — Supplementary Material 11 [file 13058_2025_2091_MOESM11_ESM.pdf]

Full uncropped Western blot images

Figure 5A

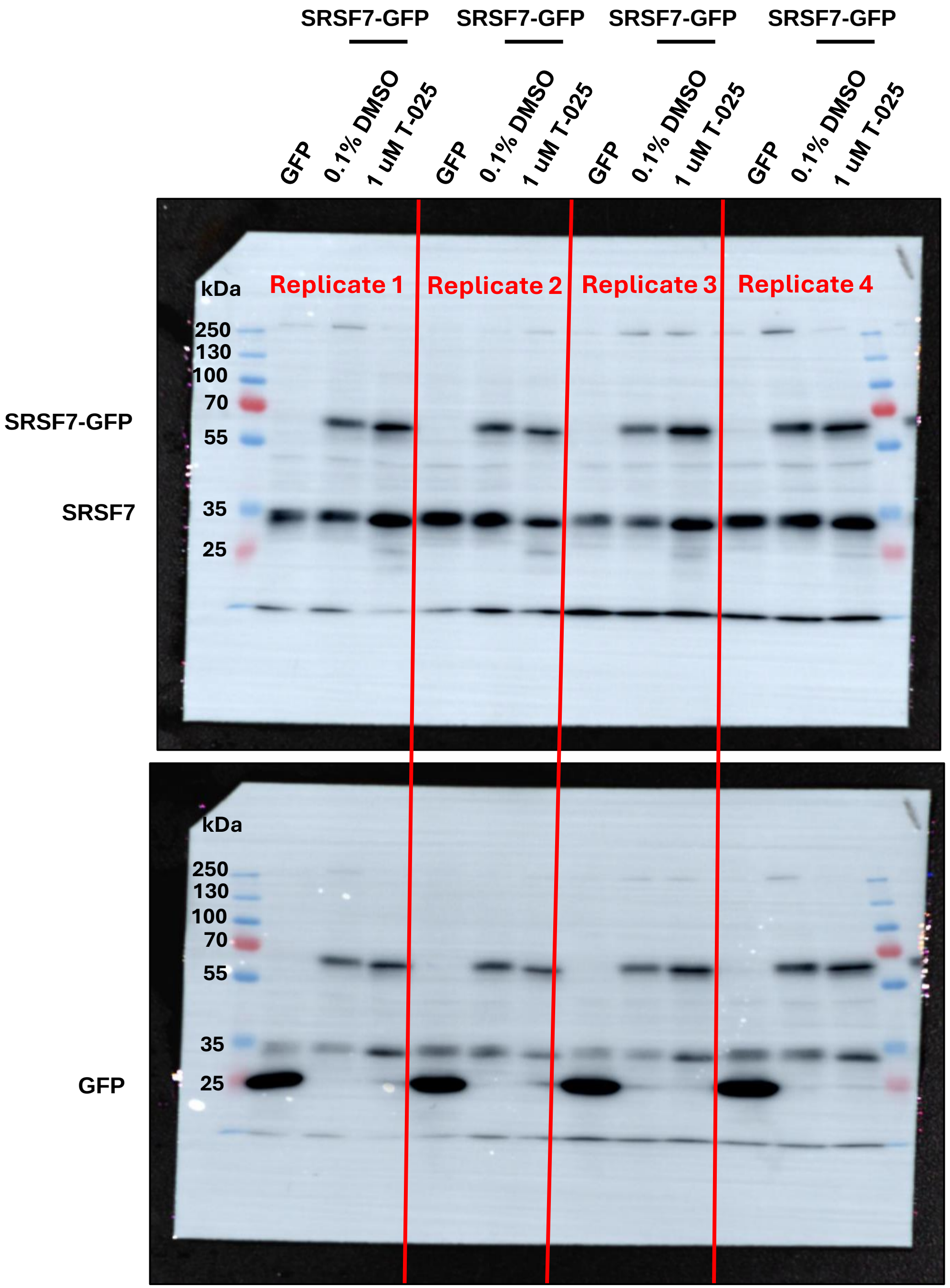

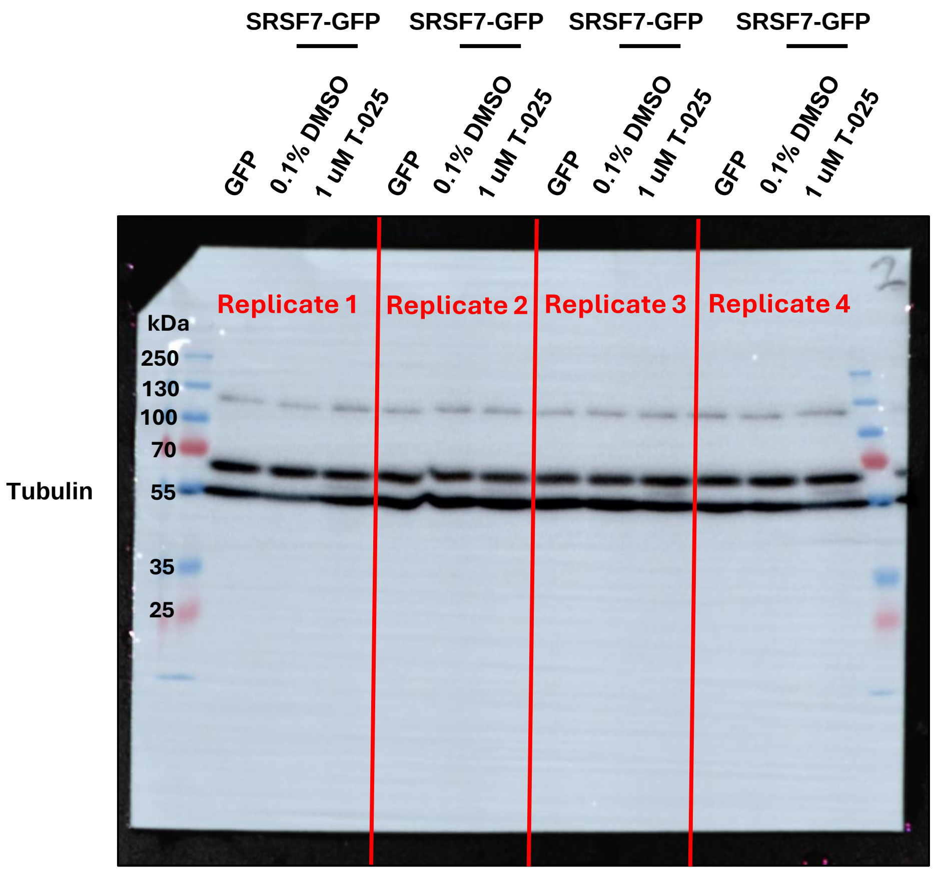

Replicate 4 was used in figure 5E-F

Figure 5E-F
Replicate 1

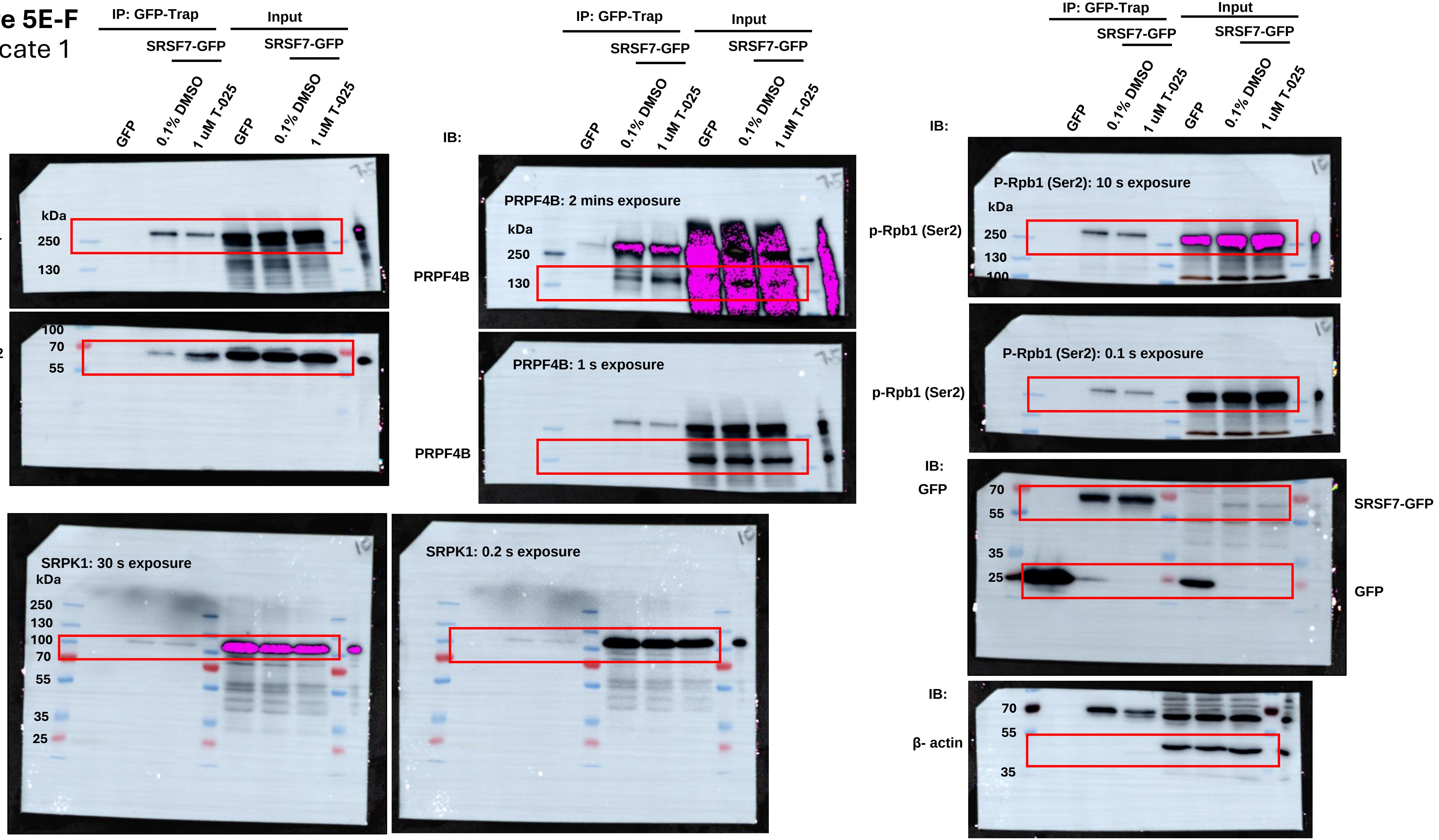

Figure 5E-F
Replicate 2

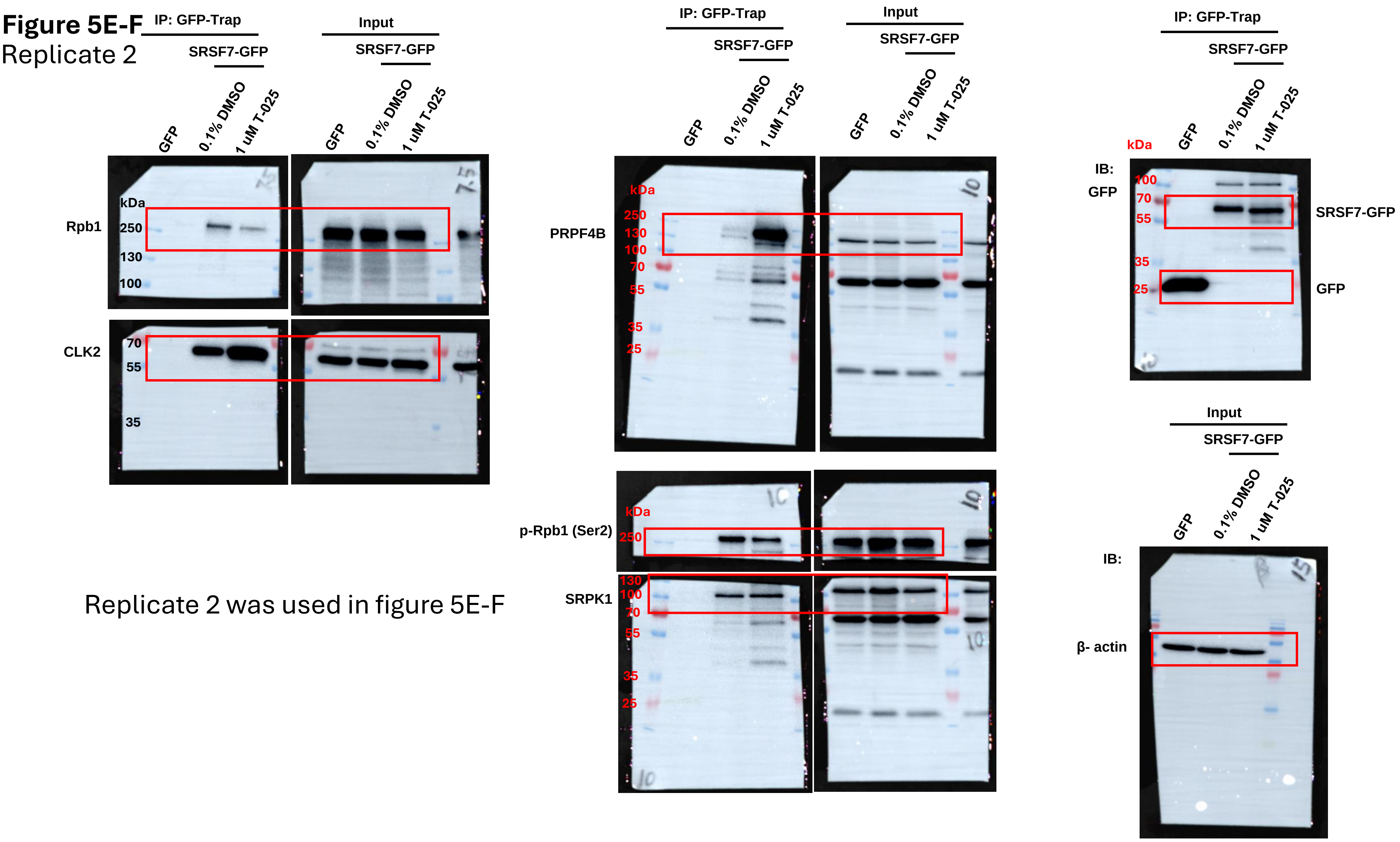

Replicate 2 was used in figure 5E-F

Figure 5E-F
Replicate 3

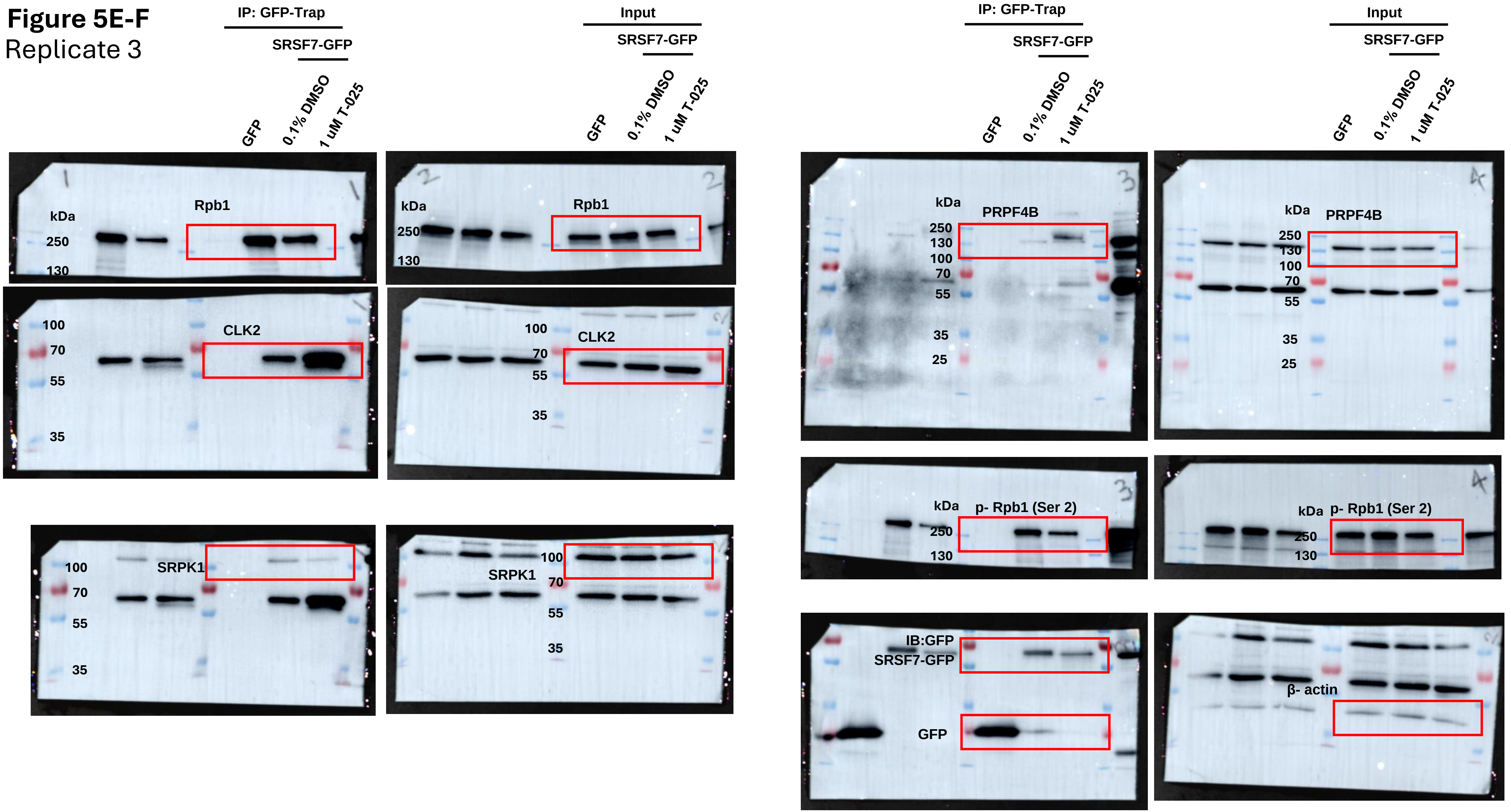

Figure 6A

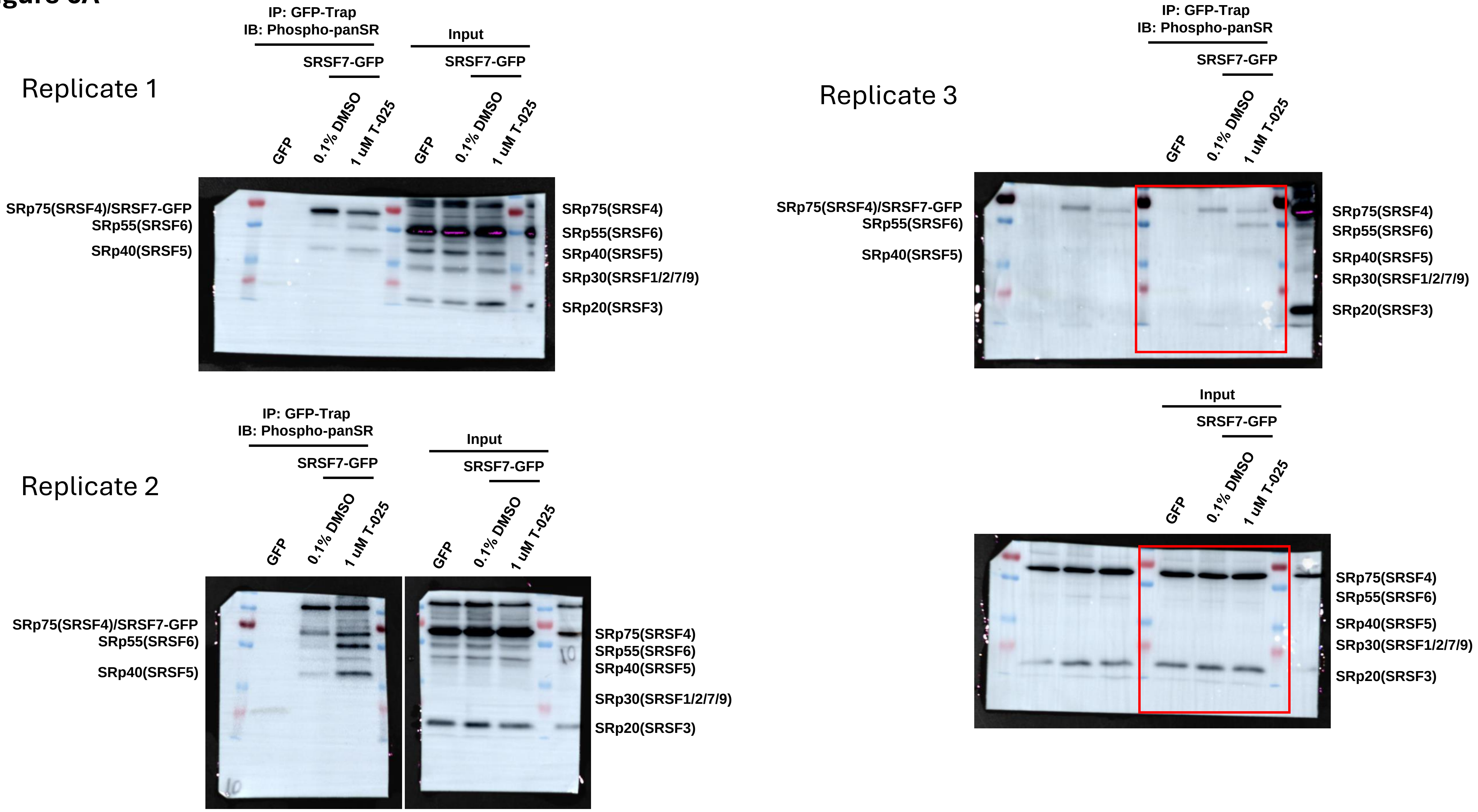
Replicate 1
IP: GFP-Trap
IB: Phospho-panSR
SRSF7-GFP
Input
SRSF7-GFP
GFP
0.1% DMSO
1 uM T-025
GFP
0.1% DMSO
1 uM T-025
SRp75(SRSF4)/SRSF7-GFP
SRp55(SRSF6)
SRp40(SRSF5)
SRp75(SRSF4)
SRp55(SRSF6)
SRp40(SRSF5)
SRp30(SRSF1/2/7/9)
SRp20(SRSF3)
Replicate 2
IP: GFP-Trap
IB: Phospho-panSR
SRSF7-GFP
Input
SRSF7-GFP
GFP
0.1% DMSO
1 uM T-025
GFP
0.1% DMSO
1 uM T-025
SRp75(SRSF4)/SRSF7-GFP
SRp55(SRSF6)
SRp40(SRSF5)
SRp75(SRSF4)
SRp55(SRSF6)
SRp40(SRSF5)
SRp30(SRSF1/2/7/9)
SRp20(SRSF3)
Replicate 3
IP: GFP-Trap
IB: Phospho-panSR
SRSF7-GFP
GFP
0.1% DMSO
1 uM T-025
SRp75(SRSF4)/SRSF7-GFP
SRp55(SRSF6)
SRp40(SRSF5)
SRp75(SRSF4)
SRp55(SRSF6)
SRp40(SRSF5)
SRp30(SRSF1/2/7/9)
SRp20(SRSF3)
Input
SRSF7-GFP
GFP
0.1% DMSO
1 uM T-025
SRp75(SRSF4)
SRp55(SRSF6)
SRp40(SRSF5)
SRp30(SRSF1/2/7/9)
SRp20(SRSF3)
